# Green production of multi-functional carbon dots from agricultural corn silk waste: application in heavy metal (Fe^3+^/Cu^2+^) fluorometric sensing and in vitro cytotoxicity

**DOI:** 10.1038/s41598-026-65469-6

**Published:** 2026-08-31

**Authors:** Dina Abdelmonsef, Sherif Hammad, Mohsen Ghali

**Affiliations:** 1 School of Basic and Applied Sciences, Egypt-Japan University of Science and Technology (E-JUST), New Borg El-Arab City, Alexandria Egypt; 2 PharmD Program, Egypt-Japan University of Science and Technology (E-JUST), New Borg El-Arab City, Alexandria Egypt; 3 Pharmaceutical Chemistry Department, Faculty of Pharmacy, Capital university (formerly Helwan University), Cairo, 11795 Egypt; 4 Physics Department, Faculty of Science, Kafrelsheikh University, Kafrelsheikh, Egypt

**Keywords:** Non-synthetic carbon dots (CDs), Corn silk (CS), Antimicrobial activity, Cytotoxicity, Fe^3+^ and Cu^2+^ sensor, Chemistry, Environmental sciences, Materials science, Nanoscience and technology

## Abstract

Corn silk (CS), an abundant agricultural waste, is typically converted into carbon dots (CDs) through energy-intensive methods such as hydrothermal treatment and microwave-assisted synthesis. In contrast, this study introduces a novel, simple, non-thermal (non-synthetic), and chemical-free extraction strategy to produce CDs directly from corn silk at ambient conditions. The properties of the resulting CDs were comprehensively characterized using transmission electron microscopy (TEM), Fourier transform infrared (FTIR) spectroscopy, and photoluminescence (PL) spectroscopy. While existing literature extensively reports biomass-derived CDs as probes for Fe^3+^ detection, their application for Cu^2+^ sensing remains limited and often requires complex surface functionalization. Remarkably, our non-synthetic extracted CDs exhibit high sensitivity toward Cu^2+^ ions with a competitive limit of detection (LOD) of 33 nM. Furthermore, to elucidate the impact of processing conditions, we systematically investigated the hydrothermal carbonization of corn silk across a temperature gradient. Our findings reveal that the preparation method acts as a functional switch: the non-synthetic route enables Cu^2+^ sensing, while hydrothermal treatment optimizes Fe^3+^ detection at 200 °C and enhances antimicrobial activity at 240 °C. This work establishes the first energy-free, non-synthetic approach to transform corn silk into high-performance Cu^2+^ nanoprobes, providing a sustainable and tunable platform for converting biomass into multifunctional nanomaterials.

**Supplementary Information:**

The online version contains supplementary material available at https://doi.org/10.1038/s41598-026-65469-6.

## Introduction

=The improper disposal of agricultural waste often leads to soil and water contamination. In recent years, there has been a growing interest in the valorization of agricultural by-products due to their abundant availability, the presence of valuable compounds, and the increasing need for more efficient and sustainable waste management in manufacturing processes^1^. In this context, the study presents an alternative approach to mitigate soil pollution through converting agricultural solid waste, such as corn silk (CS), into carbon materials.

Corn (*Zea mays*) is one of the most widely cultivated crops globally. According to the International Grains Council (IGC), global production is projected to reach 1.233 billion metric tons in the 2024/2025 marketing year, up from approximately 1.15 billion metric tons in 2023^2^. Global corn production generates significant by-products from the unused parts of the plant, including tassels, leaves, cobs, kernels, silk, and husks. Corn silk (Stigma maydis, CS) consists of the yellowish, thread-like stigmas of the female maize flower^3^. Corn silk is often used in health products due to its medicinal qualities; CS is an advantageous botanical treatment that promotes and sustains total health. It comprises many minerals, including magnesium, calcium, sodium, and potassium, along with a range of bioactive components such as vitamins, flavonoids, phenolic compounds, steroids, alkaloids, carbohydrates, and proteins^4,5^. The global corn silk production represents 100–300 million tons, and the silk by-product of corn accounts for around 3–5% of the crn plant. Consequently, this corn silk is generated annually by a huge amount from 3 to 15 million tons. That is why it will be very crucial to identify more beneficial functional compounds in maize silk that would be economically and environmentally beneficial, enhancing its commercial value and minimizing waste^6^. Owing to its abundance, affordability, and biodegradability, maize silk serves as an ideal natural precursor for the green synthesis of multifunctional carbon-based products^7–9^. This study presents a simple and novel, non-thermal extraction strategy to produce CDs from corn silk for the first time.

Carbon dots (CDs) are a novel material because of their outstanding physical and optical properties^10^. These quantum dots illustrated excitation-dependent emission. CDs are extremely small, measuring less than 10 nanometers across, making them one of the tiniest nanomaterials. Unlike traditional metal-based quantum dots, which are often toxic (such as those containing cadmium, lead, and indium), CDs generally have low toxicity and are well-suited for use in living systems. This makes them promising candidates for various medical applications including antibacterial, anticancer^11^, drug delivery, bioimaging, antiviral, and wound healing treatments^12^.

The excessive use of antibiotics accelerates the development of bacterial resistance^13^. This situation generates a critical need for novel antibacterial agents to address the threat posed by antibiotic-resistant microorganisms. Carbon dots (CDs) present a convincing choice for this application, owing to their biocompatibility, controlled structure, functional properties, and ease of synthesis^14^. However, the degree of toxicity of CDs remains an obstacle. Therefore, this research focused on generating a new antibiotic agent from corn silk-derived carbon dots with a low degree of toxicity. Numerous synthetic techniques have been established for the preparation of C-dots, including top-down methods such as laser ablation and electrochemical oxidation^15^, Bottom-up^15^ methodologies for microwave-assisted synthesis, hydrothermal production procedures^16^, and combustion thermal oxidation processes. Nevertheless, all artificial methodologies display specific drawbacks, such as extreme reaction circumstances, complex procedures, or dangerous starting materials. In this work, CDs are obtained from natural biomass sources with and without the need for chemical, mechanical, or electrochemical procedures^17^.

Heavy metals like copper (Cu), iron (Fe), zinc (Zn), and cobalt (Co) are needed in very small amounts to keep life going because they play many important roles in biology^18,19^. Copper (Cu^2+^) and ferric (Fe^3+^) ions are essential heavy metals with significant roles in environmental and public health^20^. Copper and ferric ions get involved in water and soil through industrial wastewater, mining runoff, and agricultural pollution, and can accumulate to a toxicity level in living things. Among these, the shortage of copper in the body has an impact on enzyme functions and disrupts cellular biochemical activity, leading to many neurological and blood problems such as anemia and leukoderma^21^. On the other hand, a high level of copper can cause a myriad of ailments such as gastrointestinal disorders, neurological problems, and damage to the liver and kidneys^22^. In the case of Ferric ions, Fe^3+^ is a vital player in human metabolism and is a fundamental component of various proteins. Additionally, the body’s imbalance and deficiency of Fe^3+^ causes anemia, liver damage, diabetes, cancer, and Parkinson’s diseases, excessive levels of Fe^3+^ can harm the body’s normal functions, lead to oxidative stress and hemochromatosis, and contribute to Alzheimer’s and other neurodegenerative diseases through overproduction of H_2_O_2_ from consuming tainted water containing the element^23^. So, the creation of metal ion sensors is still a challenging issue^24^. In this context, multiple analytical techniques, such as atomic absorption spectroscopy^25^, mass spectrometry^26^, electrochemistry^27^, nuclear magnetic resonance^27^, liquid chromatography^28^, colorimetry^29^, and electrochemiluminescence^30^, are currently methods that are utilized for the detection of Cu^2+^ and Fe^3+^. However, these established techniques necessitate costly reagents, intricate preparation procedures, and sophisticated, high-cost equipment for the concurrent detection of Cu^2+^ and Fe^3+^. Hence, there is a pressing need for simple, highly sensitive, and cost-effective methods, such as fluorescence-based sensors^23^.

Although biomass-derived carbon dots (CDs) are lauded for their sustainability and tunable optical properties, the field remains dominated by energy-intensive hydrothermal synthesis^31,32^. Furthermore, biomass-based CDs typically exhibit high selectivity for Fe^3+^ ions^33–35^, whereas sensitive detection of Cu^2+^ often requires complex surface functionalization^36–39^. In this work, we address this gap through a novel, non-synthetic, and non-thermal extraction strategy. This approach produces CDs that possess a high inherent nitrogen content (5.03%), derived directly from the corn silk precursor as confirmed by XPS. These CDs enable highly sensitive direct Cu^2+^ sensing with a competitive limit of detection (LOD) of 33 nM. To further elucidate the role of processing conditions, we also investigate the hydrothermal carbonization of corn silk across a temperature gradient, characterizing the resulting CDs for diverse analytical applications. Unlike previously reported corn silk-derived carbon dots, which are mainly synthesized via hydrothermal routes and show limited Cu²⁺ sensing performance, the present work demonstrates a direct extraction strategy yielding intrinsic nitrogen-doped CDs with significantly enhanced Cu²⁺ sensitivity and ultralow detection limits.

## Experimental sections

### Materials

Corn was obtained from local farmers, Alexandria, Egypt, Ultrapure water was obtained using a Milli-Q purification system. Dichloromethane, methanol, and acetone (≥ 99%, Sigma Aldrich) were used as received. Silica gel (60–120 Å) was used for column chromatography.

### Extraction of natural CDs (non-synthetic CDs)

**Fig. 1:**
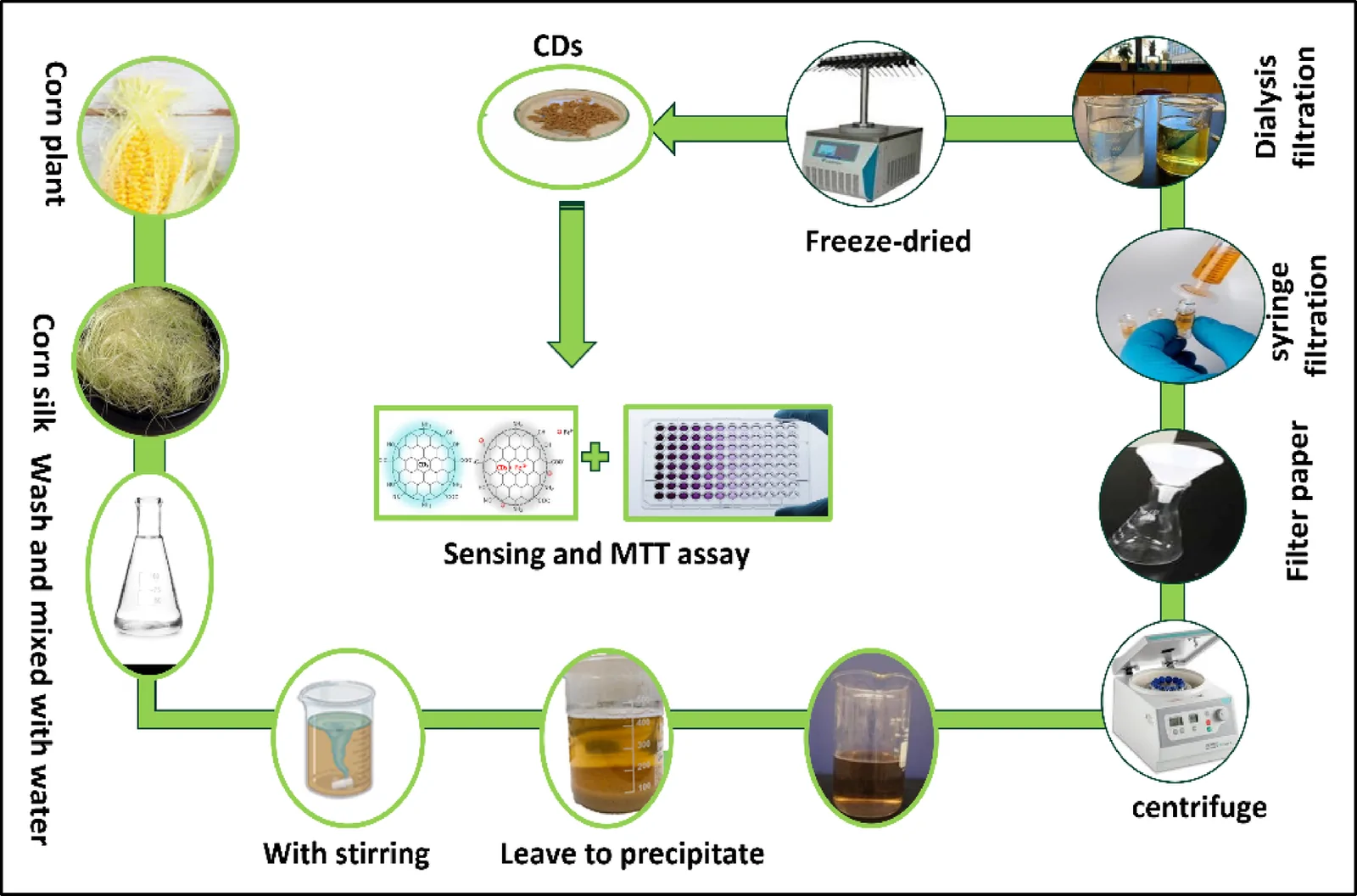
Schematic diagram of the extraction method of CDs from corn silk.

The fresh maize was obtained from a local farm. The corn silk was grasped from the corn plant. Corn silk was totally rinsed with fresh water to remove impurities and subsequently chopped. The chopped corn silk was mixed with deionized water and thoroughly stirred to stimulate the extraction of carbon dots (CDs). The mixture is left for three weeks, till the solution exhibits a cyan emission when exposed to UV light with a wavelength of 395 nm. The resultant solution undergoes some purification methods to isolate the CDs. First, the solution was further filtered by centrifugation at 6000 rpm for 30 min and then through a 0.22 μm Whatman filter paper to exclude the majority of bulk materials mixed with the aqueous solution. Subsequently, the solution was dialyzed through a membrane (MWCO 1KDa) for 48 h to exclude carbohydrates and free chemicals that could induce luminescence quenching. After freeze-drying, the CD powder was obtained Fig. 1.

### Synthesis of CDs

**Fig. 2:**
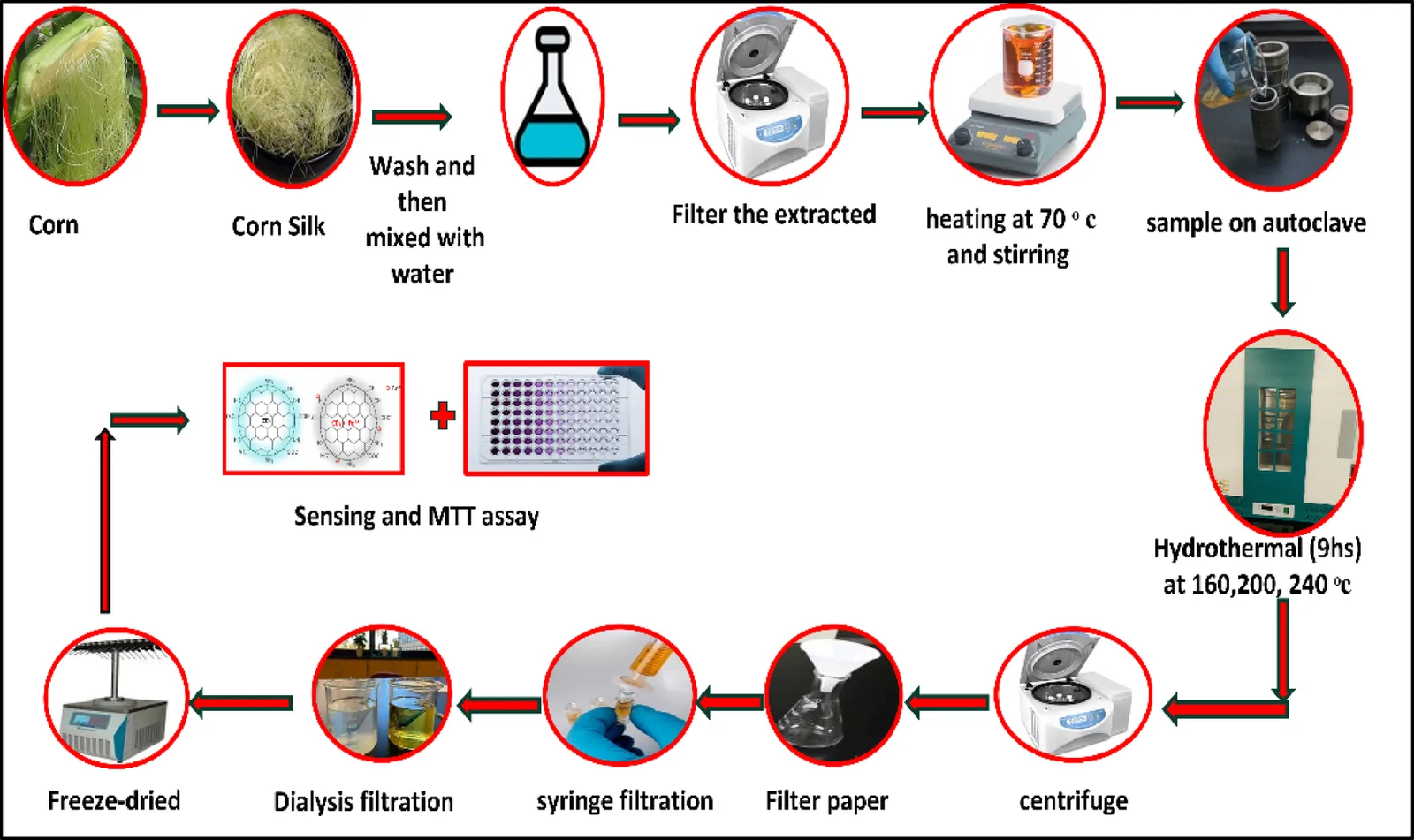
The schematic diagram indicates CDs synthesis from Corn silk.

The fresh corn was procured from a local farmer. Corn silk was meticulously rinsed with deionized water and subsequently chopped. The bits of corn silk were mixed with deionized water. Subsequently, 45 mL of the solution was subjected to heating and agitation at 69 °C for 20 min. This solution was subsequently subjected to hydrothermal treatment at different temperatures (160,200,240 °C) for 9 h in a furnace, forming CDs-160, CDs-200, and CDs-240, respectively. A yellow solution was acquired after cooling the system, followed by filtration with filter paper (pore size = 125 mm) and centrifugation at 6000 rpm for 30 min to isolate the less soluble deposit. The CDs solution was dialyzed using 300 ml of deionized water. Approximately 3 ml of the crude product was isolated using column chromatography utilizing a column with a fused-in frit of 500 × 20 mm. The powdered CDs were obtained by freezing-drying for about 60 h as shown in Fig. 2.

### Plausible mechanism for producing CDs

**Fig. 3:**
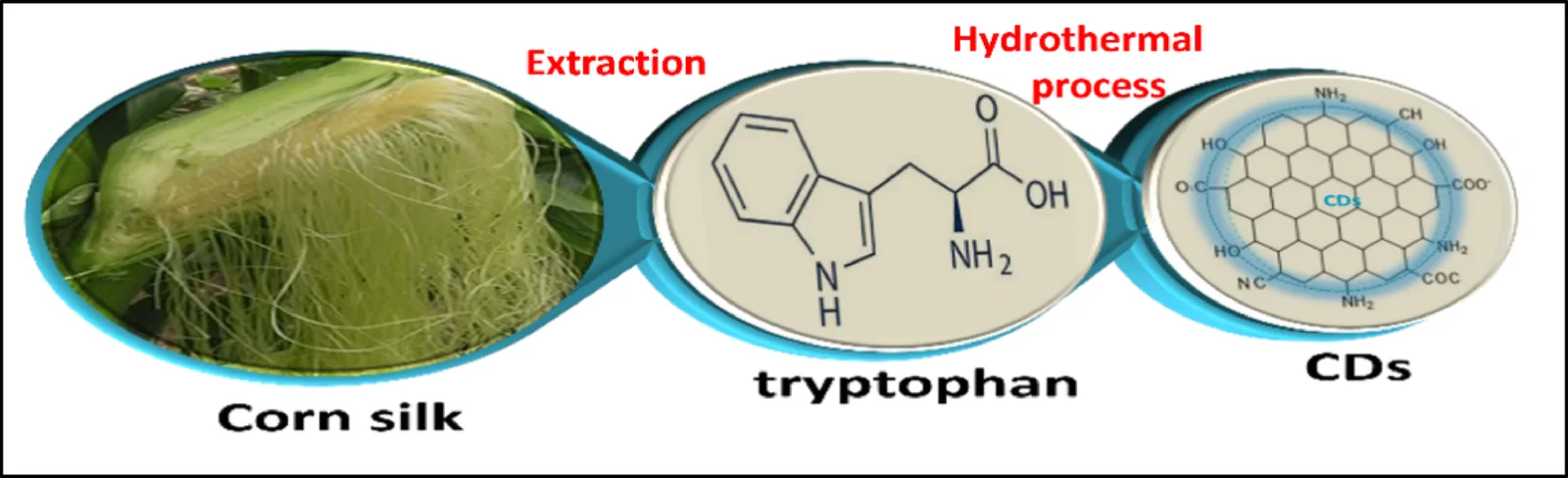
A simple schematic diagram demonstrates the synthesis of CDs from corn silk.

The corn silk was further divided into four primary fractions according to their distinct solubility properties, utilizing the Osbourne technique: albumins (soluble in water), globulins (soluble in salt), glutelins (soluble in alkaline solutions), and prolamins (soluble in alcohol). Albumin comprises important amino acids, including lysine and tryptophan. As tryptophan could be the bioactive for producing CDs, Tryptophan is a significant intrinsic fluorescent probe. It contains an α-carboxylic acid group, an α-amino group, and an indole side chain, thereby categorizing it as a non-polar aromatic amino acid^40,41^. Most likely, the heating temperature at high temperature and the pressure inside the autoclave can hydrolyze tryptophan and break these labile oxygen and hydrogen linkages. Therefore, the reaction proceeds by the breaking of the labile bonds, followed by the release of H_2_O and then the subsequent graphitic leading eventually to the creation of carbon rings as illustrated in Fig. 3.

### Characterization

The shape of particles, their size, and structure were analyzed by the utilization of a high-resolution transmission electron microscope (TEM), JEM-2100 F from JEOL Company, equipped with a 200 kV field emission revolver. The Fourier Transform Infrared spectra (FTIR) analysis was achieved using a Bruker Invenio S Fourier FT-IR spectrometer. The crystal structure was studied by an X-ray diffractometer 6100 (XRD) from Shimadzu Company, with a wavelength of 1.546 Å. At the same time, a Thermo Fisher Scientific K-Alph X-ray photoelectron was used to illustrate XPS spectra. UV clarified the optical properties–Visible absorption spectra were established using the Hitachi U-3900 UV spectrophotometer, and Photoluminescence measurements were made using a Shimadzu RF-6000 fluorescence spectrometer. The absolute PL quantum yield (QY) was studied by utilizing an FS5 spectrofluorometer with an integrating sphere; meanwhile, the time-resolved fluorescence decay was conducted by a spectrophotometer that contains a Time-Correlated Single Photon Counting (TCSPC, Horiba, FS5) with a 340 nm pulsed LED light source. A Zeta sizer (Malvern Zeta sizer Nano Series) was applied to determine the stability and the net charge of CDs.

## Antimicrobial activity

### In vitro antimicrobial activity test

The antibacterial activity of the CDs was assessed using a disc diffusion assay^42^. This approach was employed to analyze six pathogenic organisms: two gram-positive, two gram-negative, and two fungal strains. The Gram-negative bacterial strains were cultivated on Mueller-Hinton agar (MHA). MHA plates were incubated at 37 °C, while the fungi were cultivated in Sabouraud Dextrose Agar (SDA) and Potato Dextrose Agar (PDA) at 28 °C. CDs of two dilutions (5 mg/mL and 100 mg/mL) were produced and applied to sterile discs on the surface of MHA plates. The sensitivity of the pathogenic microorganisms to CDs were evaluated by Inhibition Zone (ZOI), with the incubation process carried out at 37 °C for 18 to 24 h for bacteria and at 28 °C for 48 h for fungi^43^. Gentamicin was utilized as a standard medication for Gram-positive and Gram-negative bacteria. Fluconazole served as the reference medication for fungal strains.

### MIC test

The following strains were used for the test: Staphylococcus aureus, Bacillus subtilis (ATCC 6633), Escherichia coli (ATCC 8739), Klebsiella pneumoniae (ATCC 13883), and Candida albicans (ATCC 10221). TCDs were serially diluted in Tryptic soy broth within a 96-well microtiter plate to achieve concentrations from 1000 µg/mL down to 3.9 µg/mL. Each well was inoculated with 100 µL of microbial suspension. A blank well containing only the medium and a positive control well containing microorganisms, but no CDs were included. A 20-hour incubation period was performed at 37 °C. After incubation, a BioTek 800 TS microplate reader was used to measure at 630 nm the optical density (OD). The MIC value was clarified as the minimum concentration of CDs that inhibited the growth of the microorganisms^44^.

## Cytotoxicity test

### Cell culture

MCF-7, a mortal breast cancer cell line from the ATCC, was cultivated in RPMI-1640 medium. This medium was enriched with 10% inactivated fetal calf serum and 50 µg/ml gentamycin. The cell line was kept at 37 °C in a humidified 5% CO2 environment and was sub-cultured two to three times a week.

### Cell viability assay

The cytocompatibility of the compounds was assessed using the MTT test, which was performed in agreement with established methods^45^. For antitumor assays, MCF-7 cells were suspended in medium at a concentration of 1 × 10^5^ cells/well in Corning^®^ 96-well tissue culture plates and then incubated for 24 h. The tested compounds, dissolved in Phosphate-buffered saline (PBS), were added to 96-well plates (three replicates) to achieve eight different concentrations for each compound (7.8, 15.6, 31.25, 62.5, 125, 250, 500, 1000 µg mL^− 1^). Six vehicle controls with media were run on each 96-well plate. After incubating for 48 h, the MTT assay was used to count the number of living cells. The procedure involved a brief process of replacing the old media with fresh RPMI 1640 medium and adding a 12 mM MTT stock solution to each well. After this, the plates were incubated for four hours under specific conditions (37 °C and 5% CO_2_). After removing an 85 µL aliquot of the media from each well, 50 µL of DMSO was added, which was mixed with a pipette. After a 10-minute incubation at 37 °C, the optical density was estimated at 590 nm with the microplate reader model (SunRise, TECAN, Inc, USA) to determine the number and percentage of viable cells. The following equation was used to compute the percentage of cell viability:

1 $$\:\mathrm{c}\mathrm{e}\mathrm{l}\mathrm{l}\:\mathrm{v}\mathrm{i}\mathrm{a}\mathrm{b}\mathrm{i}\mathrm{l}\mathrm{i}\mathrm{t}\mathrm{y}\:\left(\mathrm{\%}\right)\:=\frac{{OD}_{treated}}{{OD}_{control}}\times\:100\:\%$$

OD_treated_ is the mean optical density of wells treated with the tested sample and OD_control_ is the mean optical density of untreated cells. The relation between surviving cells and drug concentration was plotted to create a survival curve for each tumor cell line. The 50% inhibitory concentration (IC_50_), defined as the concentration required to cause toxic effects in 50% of intact cells, was estimated from the dose-response curve using GraphPad Prism software (San Diego, CA, USA).

### Statistical analysis

All analyses, with the exception of instrumental characterization, were conducted in triplicate at a minimum. Statistical analysis was performed using GraphPad Prism software, version 8.01 (GraphPad Software, USA). Data was analyzed using Analysis of Variance (ANOVA) followed by Duncan’s multiple range test at a significance level of *p* < 0.05. Results are presented as the mean ± standard deviation.

## Results and discussion

### Structural properties

#### TEM analysis

**Fig. 4:**
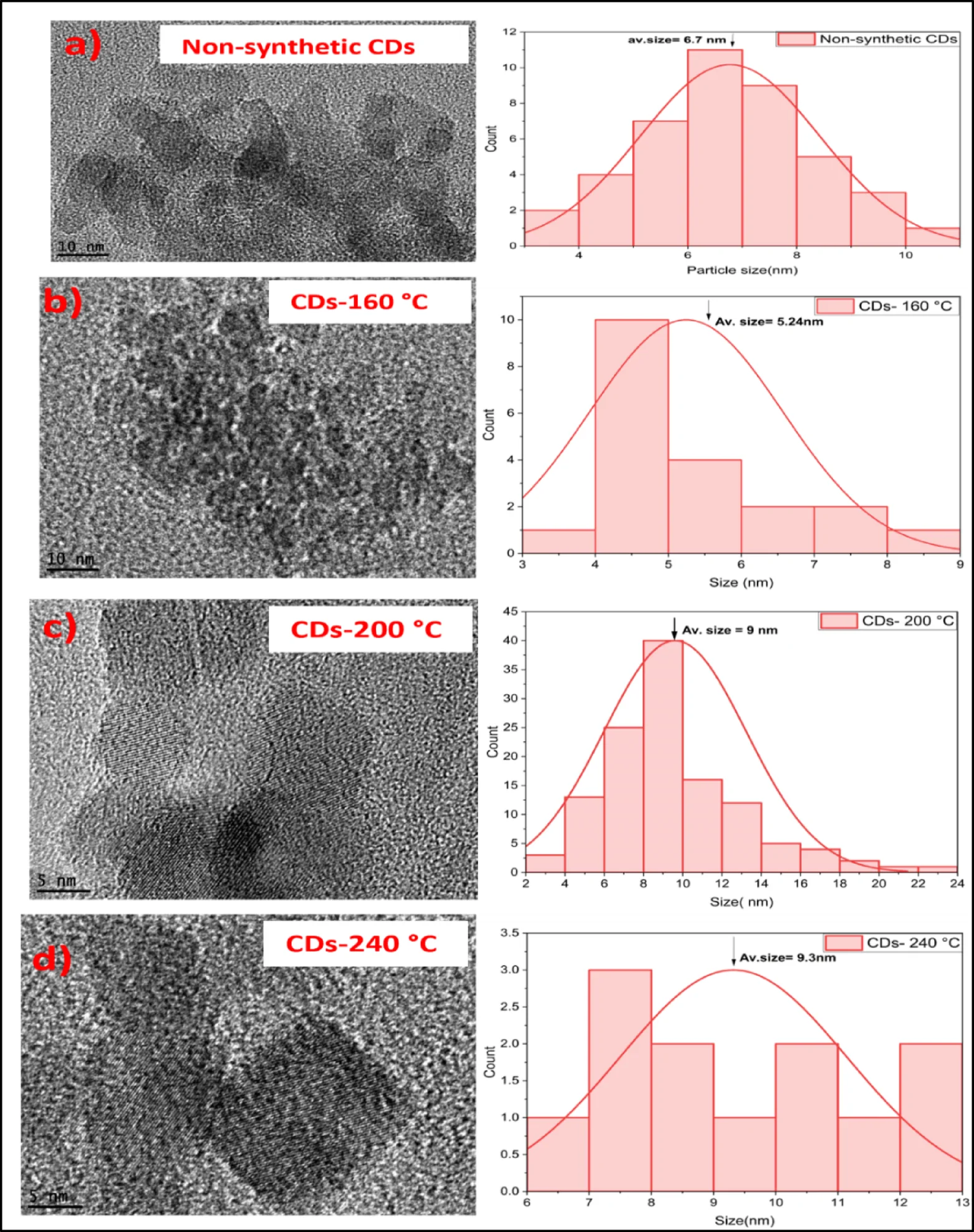
The TEM image of non- synthetic CDs (**a**) and synthetic CDs prepared at 160 °C (**b**), 200 °C (**c**) and 240 °C (**d**) with the distribution size.

Transmission electron microscopy (TEM) investigations were performed to elucidate the morphological structure of the produced carbon dots. The TEM pictures in Fig. 4 demonstrate the presence of dark spots, confirming the natural existence of CDs in corn silk, as illustrated in Fig. 4a, and the presence of synthesized CDs at temperatures of 160 °C, 200 °C, and 240 °C. They have a quasi-spherical morphology with average dimensions of 6.7 nm, 5.24 nm, 9 nm, and 9.3 nm. Non-synthetic CDs, originating from large precursors found in corn silk, exhibit a relatively large initial size of 6.7 nm. Upon heating to 160 °C, the size decreases to 5.24 nm because the thermal energy breaks H and O bonds, fragmenting the large precursor molecules into smaller pieces^16^; however, this temperature is insufficient for full carbonization. At 200 °C, the thermal energy becomes sufficient to trigger pyrolysis, dehydration, and complete carbonization, allowing these fragments to reorganize into well-defined, spherical CDs with an increased average size of 9 nm. Further increasing the temperature to 240 °C leads to even higher levels of carbonization and particle aggregation, resulting in a final average size of 9.3 nm.

#### XRD analysis

**Fig. 5:**
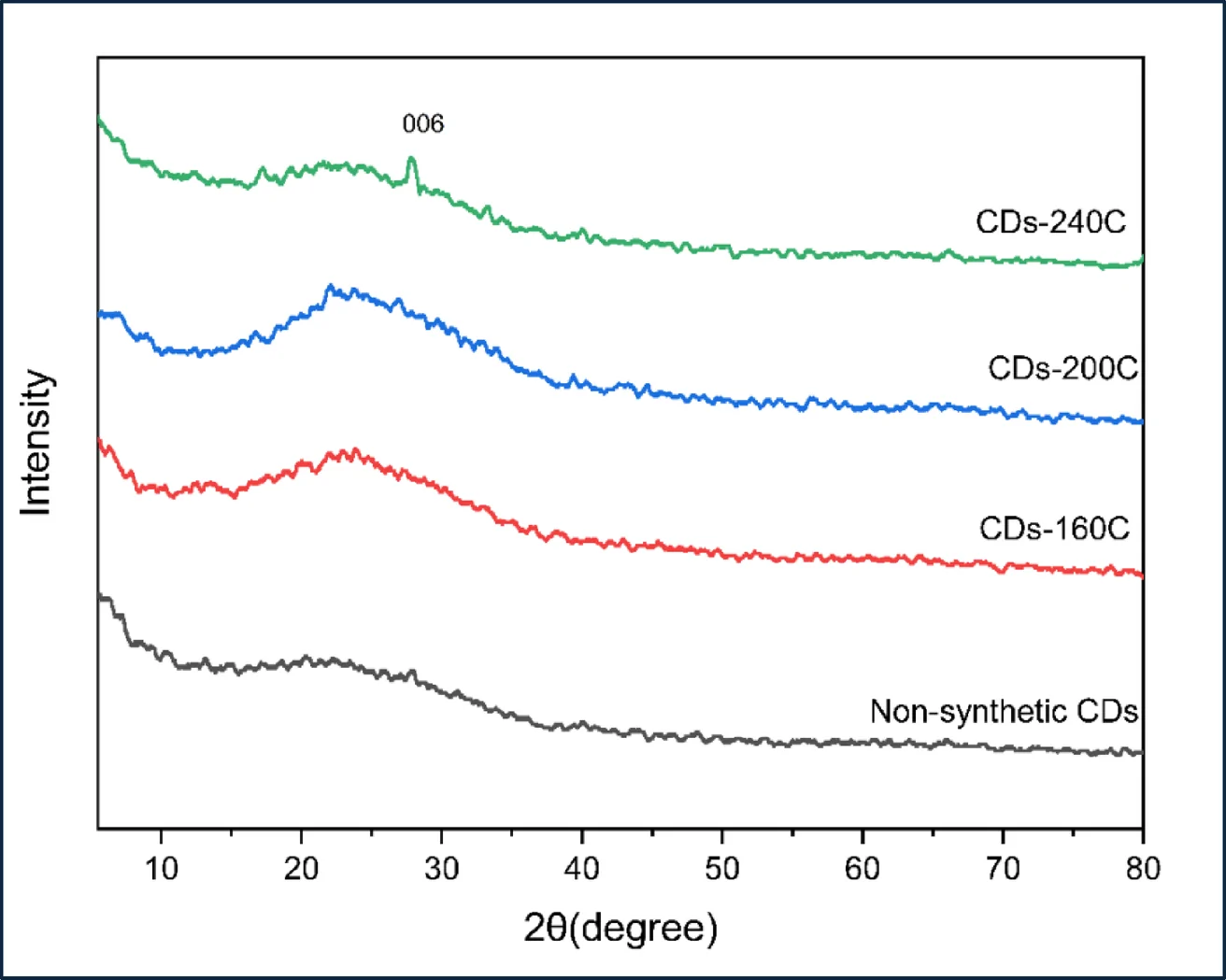
XRD of non-synthetic and synthetic CDs prepared at temperatures 160, 200 and 240 °C.

An X-ray diffraction analysis was conducted to examine the crystal structure of the non-synthetic and synthesized CDs. Figure 5 illustrates the XRD patterns of CDs synthesized at hydrothermal treatment temperatures of 160, 200, and 240 °C and non-synthetic CDs. The results demonstrate that the XRD pattern of CDs exhibited a broad diffraction peak at approximately 2θ = 24°, which corresponds to the (002) plane^46,47^. A pronounced peak at about 2θ = 27.3° can be attributed to the (006) plane in the XRD pattern, with a d spacing of 0.32 nm^48^, found in samples treated at 240 °C. This suggests that CDs exhibit a degree of crystallinity as the temperature increases.

#### FTIR analysis

**Fig. 6:**
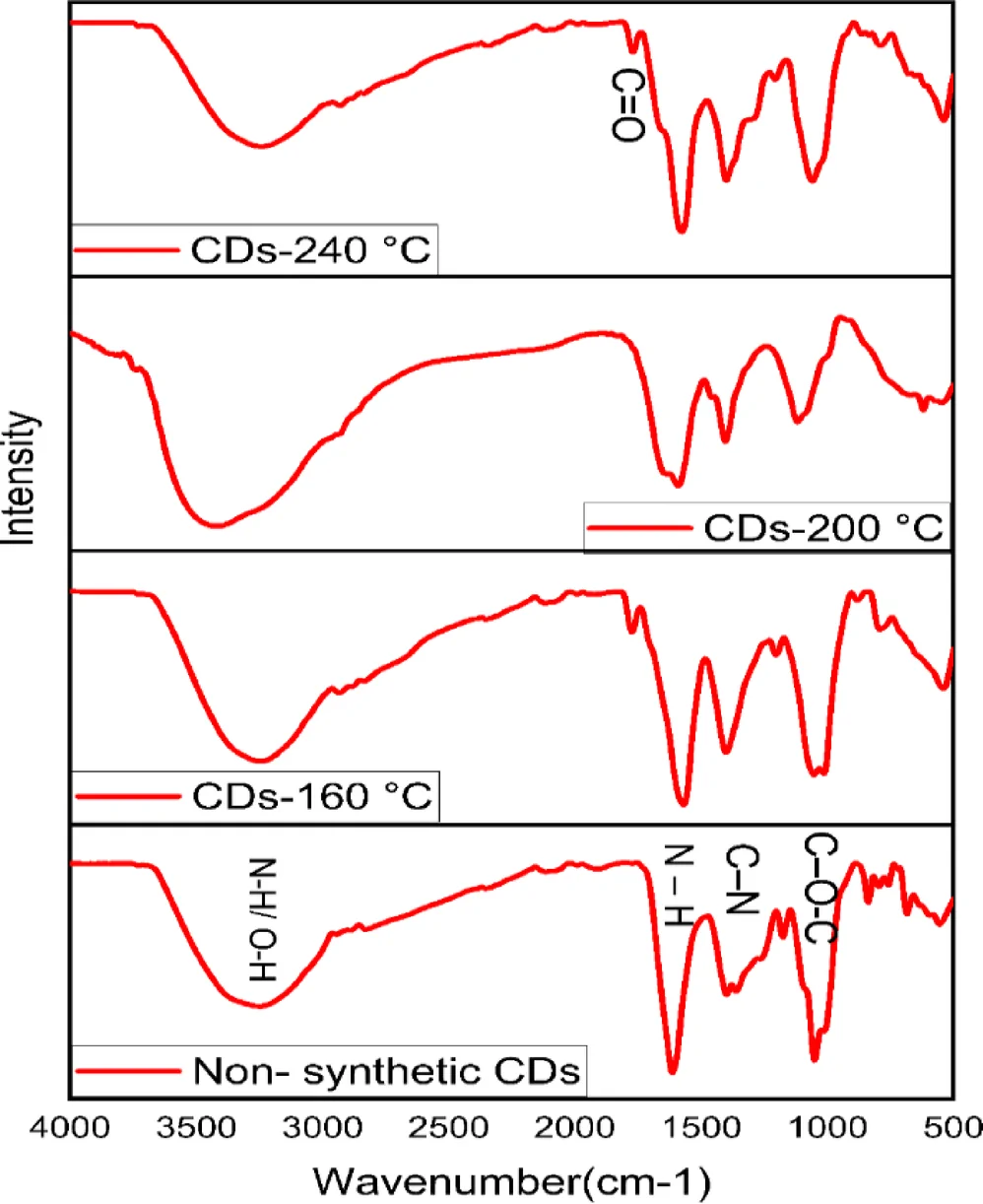
FTIR non-synthetic and synthesized CDs at different temperatures (60, 200 and 240 °C)..

The presence of functional groups on CDs was analyzed by FT-IR measurement. Figure 6 shows the FT-IR spectrum of the CDs. As noticed, a broad peak was observed at around 3500–3000 cm⁻¹ indicating O–H and N–H stretching vibrations^49^. Absorptions at 3000–2800 cm⁻¹ are due to sp³ C–H stretching^50^. N–H bending and C–N stretching vibrations appear at 1600–1500 cm⁻¹^51^. The bands in the range 1400–1200 cm⁻¹ in the amide III region are due to stretching vibrations of C–N bonds and N–H bonds, as well as the interaction of the proteins with polyphenols or polysaccharides. The transmission band at 1048 cm⁻¹ indicates C–O–C stretching vibrations of ether ester groups^6,52^. The peaks between 900 and 680 cm⁻¹ signify C–H bending in CDs^53^. Notably, no prominent C = O peak is present in non-synthetic CDs, but a new peak at 1653 cm⁻¹ assigned to conjugated or amide-type carbonyls formed at high temperature^54^ indicating that new oxygen-containing functional groups were formed on the CDs surface during the hydrothermal process. This peak is not clearly resolved in the 200 °C sample. The extensive hydrogen bonds created by oxygen-containing functional groups in raw materials such as corn silk create a complex hydrogen-bonding network, which makes it hard to analyze using FTIR, specifically the detection of C = O stretching, difficult due to peak shifting and masking by the overwhelming O-H signal. The high temperatures of the hydrothermal process help resolve this by breaking the H-bonds and allowing C = O stretching vibration in the final carbon product.

#### XPS analysis

**Fig. 7:**
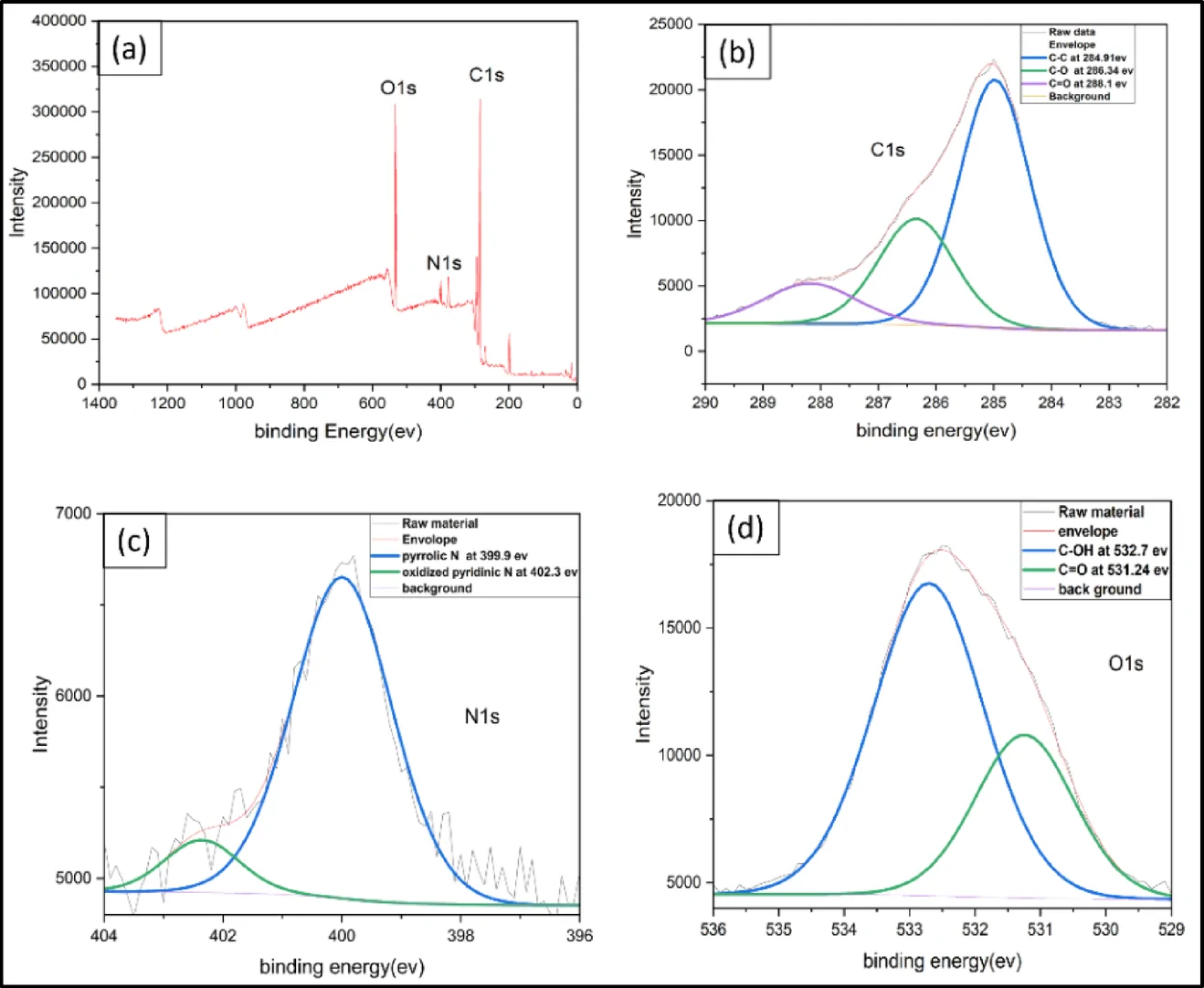
The XPS spectra of non-synthetic CDs.

**Fig. 8:**
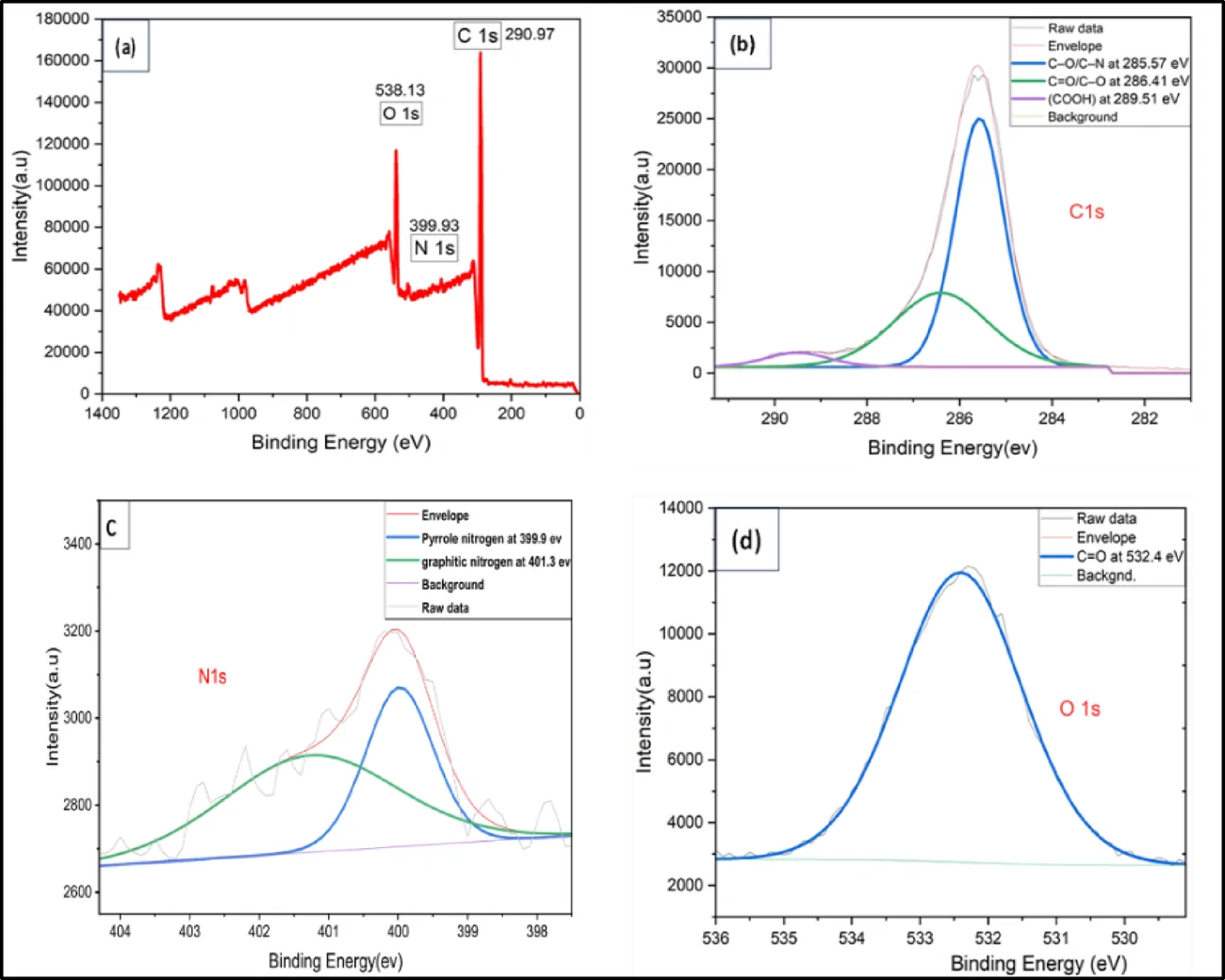
XPS spectra of CDs-200 °C.

The chemical composition of CDs was determined using the XPS technique; the complete XPS spectrum (see Figs. 7 and 8) showed that carbon makes up the majority of CDs, with nitrogen and oxygen atoms incorporated into the structure, either through doping or decoration. The XPS survey spectrum of the non-synthetic CDs shows prominent peaks corresponding to C 1s (285.48 eV, 64.88%), O 1s (532.66 eV, 21.17%), and N 1s (400.29 eV, 5.03%) (Fig. 7a). In addition, minor peaks assigned to K 2p (294.09 eV, 4.55%), Cl 2p (199.84 eV, 3.44%), and P 2p (133.83 eV, 0.92%) were also observed, which are attributed to residual inorganic species derived from the corn silk precursor^55,56^. The C 1s spectrum was deconvoluted into three peaks at 284.91 eV, 286.34 eV, and 288.18 eV, corresponding to C–C, C–O, and C = O groups (see Fig. 7b). The N1s spectra (Fig. 7c) of CDs have peaks at 399.9 eV and 402.3 eV, attributable to pyrrolic N and oxidized pyridinic N^57^, respectively indicating nitrogen atoms in the CDs. The O1s spectrum exhibits significant peaks at 531.24 eV and 532.7 eV attributable to C = O and C–OH bands as shown in Fig. 7d.

XPS measurements were performed, as seen in Fig. 8, to determine the elemental composition and precise quantities of components in the synthesized CDs at a temperature of 200 °C. No other trace of any other residual element was detected, which highlights the purity of the CDs. The XPS survey reveals the presence of Carbon (C 1s), Oxygen (O 1s) and nitrogen (N 1s) (see Fig. 8a). Oxygen and nitrogen make up a noticeable portion of the sample, with 16.05% and 0.28% respectively, while carbon makes up 83.67%. The C 1s spectrum shows peaks at 285.57, 286.41, and 284.6 eV, which correspond to C–O/C–N (60.77%), C = O/C–O (34.77%) and C-C/C = C (4.47%) bonds (Fig. 8b). The O 1s spectrum can be deconvolved into peaks at 530.89, 532.15 and 533.19 eV, representing C-O, C = O and C-OH, respectively (Fig. 8d). The N 1s spectrum was accurately matched with characteristic peaks for the pyrrolic N bond (399.9 eV) and graphitic nitrogen species (401.3 eV), demonstrating the incorporation of nitrogen elements in the carbon dots (Fig. 8c). Observations from the C 1s high-resolution XPS spectra reveal an increase in C content and peak intensity at slightly higher binding energies (285.5 eV) with a high temperature (synthesized CDs), indicating pronounced carbonization. This signifies that fast dehydration–condensation reactions of corn silk facilitated the formation of an aliphatic sp^3^ carbon structure as summarized in Table 1.

**Table 1:** Detailed XPS analysis (based on narrow scan) of the C 1s, N1s and O1s peak in non-synthetic and synthetic CDs *−* 200 °C.

	Non-synthetic CDs				Synthetic CDs- 200 °C			
	Component/assignment	Atomic %	Binding energy (eV)	FWHM (eV)	Component/assignment	Atomic %	Binding energy (eV)	FWHM (eV)
C 1s	C-O	28.99	286.34	1.45	C-O/ C-N	13.30	285.57	1.37
	C = O	6.15	288.10	1.45	C = O	4.80	286.41	1.37
	C-C	64.86	284.91	1.45	C-C/ C = C	81.90	284.6	1.37
N 1s	Pyrrole N	83.33	399.90	1.80	Pyrrole N	68.95	399.90	1.40
	Oxidized pyridinic N	16.67	402.30	1.80	Graphitic	31.05	401.30	1.40
O1s	C = O	39.30	531.24	1.68	C = O	59.86	532.15	1.47
	C-OH	60.70	532.70	1.68	C-OH	30.03	533.2	1.47
	–	–	–		C-O	10.11	530.9	1.47

#### NMR analysis

**Fig. 9:**
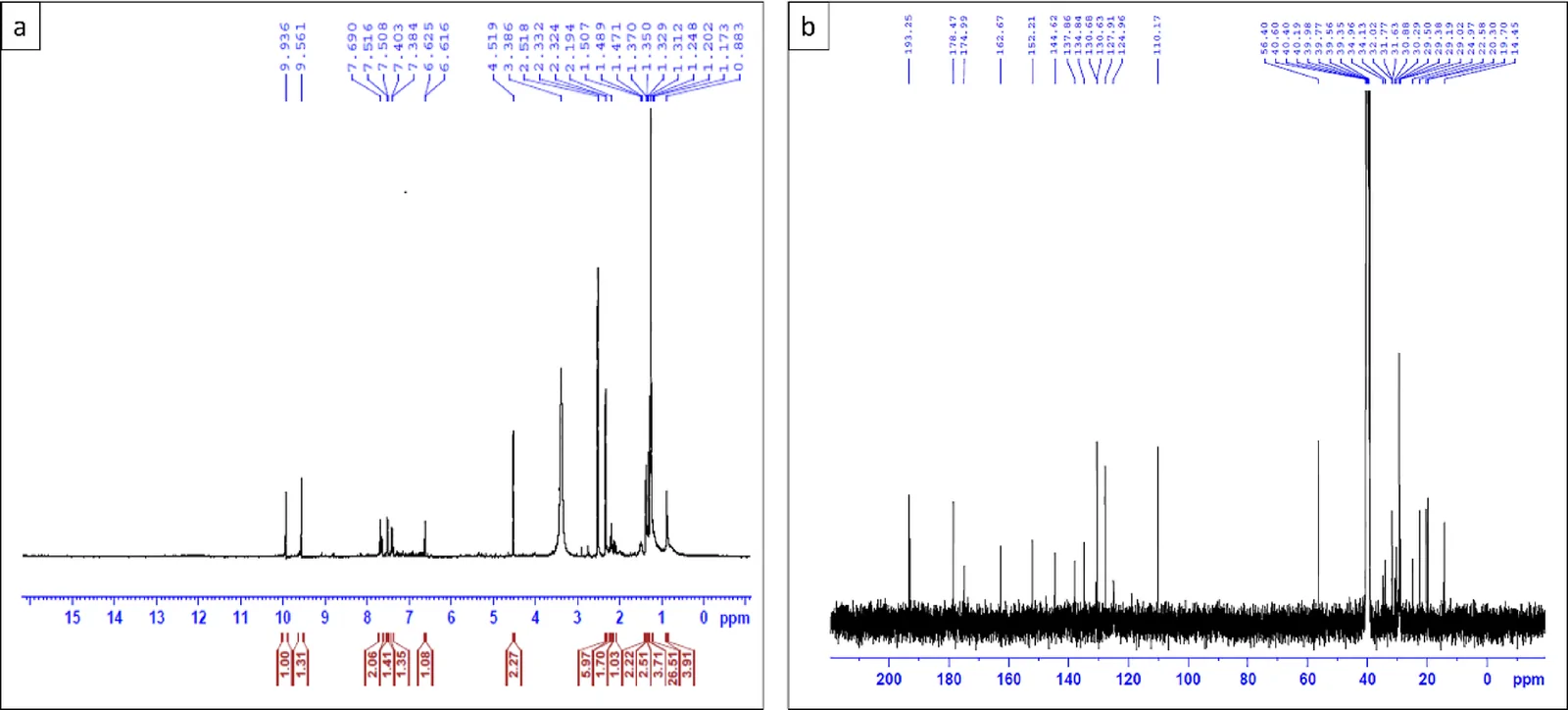
(**a**) ^1^H NMR Spectrum for CDs-200 °C and (**b**) ^13^C NMR spectra.

NMR measurements have been conducted, where NMR spectroscopy is employed to investigate the structural characteristics of the material, since they may be used to understand the hybridization that occurs in the crystalline lattices of carbon atoms. ¹H NMR results revealed the presence of sp^3^, but this is not sufficient because graphene quantum dots and carbon quantum dots include sp^3^. Additionally, they possess approximately the same size and d-spacing of planes. The type of hybridization carbon sp^2^ and sp^3^ is identified by the ^13^C NMR^58^. As shown in Fig. 9, the functional groups are mostly found in the range of sp^3^, and this is what distinguishes CDs. ^1^ H NMR spectrum (Fig. 9a) reveals the regions found are: 1–3 ppm for sp^3^ C–H protons, 3–6 ppm for protons connected with hydroxyl, ether, and carbonyl groups, 6–8 ppm for aromatic or sp^2^ protons, and 8–10 ppm for aldehydic protons. The higher intensity of peaks within sp^3^ area (aliphatic region) are more prominent than sp^2^ (aromatic region). This suggests that sp^3^ hybridized carbons dominate over sp^2^ hybridized carbon atoms. Also, in the ^13^C NMR spectrum(Fig. 9b), four similar regions: 20–60 ppm (for sp^3^ carbons and carbons attached with hydroxyl groups), the peaks in the range (80– 00) ppm can be attributed to carbons attached with ether linkages), the peaks at 100–120 ppm refer to sp^2^ carbons and 175–190 ppm are observed. Based on the increasing intensity of peaks in the sp^3^ or aliphatic protons and the number of peaks in the range sp^3^ carbon (aliphatic region), we confirm that the as-prepared particles are CDs.

#### Zeta potential analysis

**Fig. 10:**
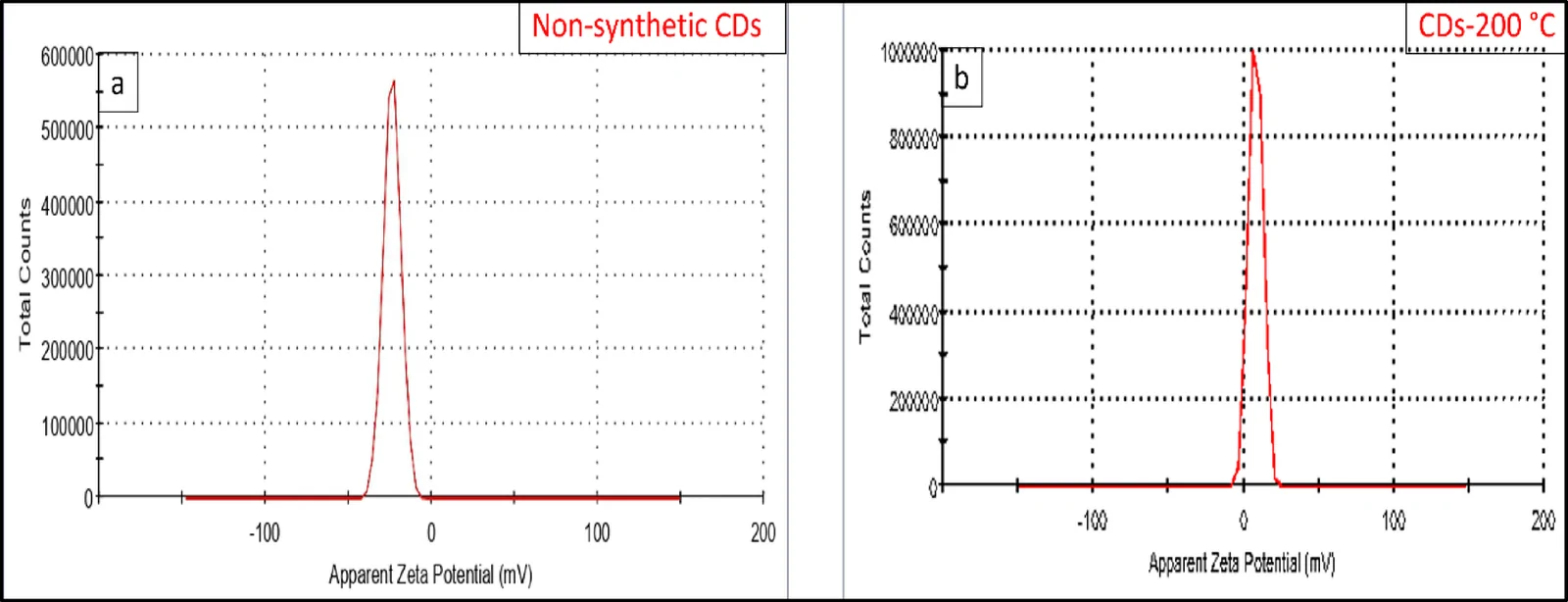
Zeta potential of non-synthetic CDs and synthetic CDs prepared at 160, 200, and 240 °C.

The non-synthetic CDs exhibited a negative zeta potential of − 25.6 mV (see Fig. 10a), indicating moderate colloidal stability. This surface charge arises from the high oxygen content (21.17%) and the presence of oxygen-containing functional groups (O–H and C–O–C)^59^, as confirmed by FTIR and XPS analyses, which undergo partial deprotonation in aqueous media. In contrast, A clear temperature-dependent increase in negative zeta potential is observed for the synthetic CDs, shifting from − 21.9 mV at 160 °C to − 25.2 mV at 200 °C and reaching − 33.4 mV at 240 °C as shown in Fig. 10. The synthetic CDs show a clear temperature-dependent increase in negative zeta potential, shifting from − 21.9 mV at 160 °C to − 25.2 mV at 200 °C and reaching − 33.4 mV at 240 °C as shown in Fig. 10. This enhancement reflects a higher degree of surface oxidation, leading to the formation of oxygenated functionalities capable of deprotonation in aqueous media, which is responsible for the enhanced negative zeta potential observed in the synthetic CDs. Dynamic light scattering (DLS) analysis was performed on all samples, including the non-synthetic CDs, CDs-160 °C, CDs-200 °C, and CDs-240 °C, to evaluate their hydrodynamic size distribution and dispersion behavior in solution (see Fig. S1). The DLS measurements revealed average hydrodynamic diameters of approximately 767 nm, 698 nm, 790 nm, and 841 nm, respectively. These relatively large hydrodynamic sizes are attributed to concentration-dependent interparticle interactions and partial aggregation in the aqueous medium^60^, since DLS measures the hydrodynamic diameter rather than the core particle size.

### Optical properties

#### UV–visible and PL spectrum analysis

**Fig. 11:**
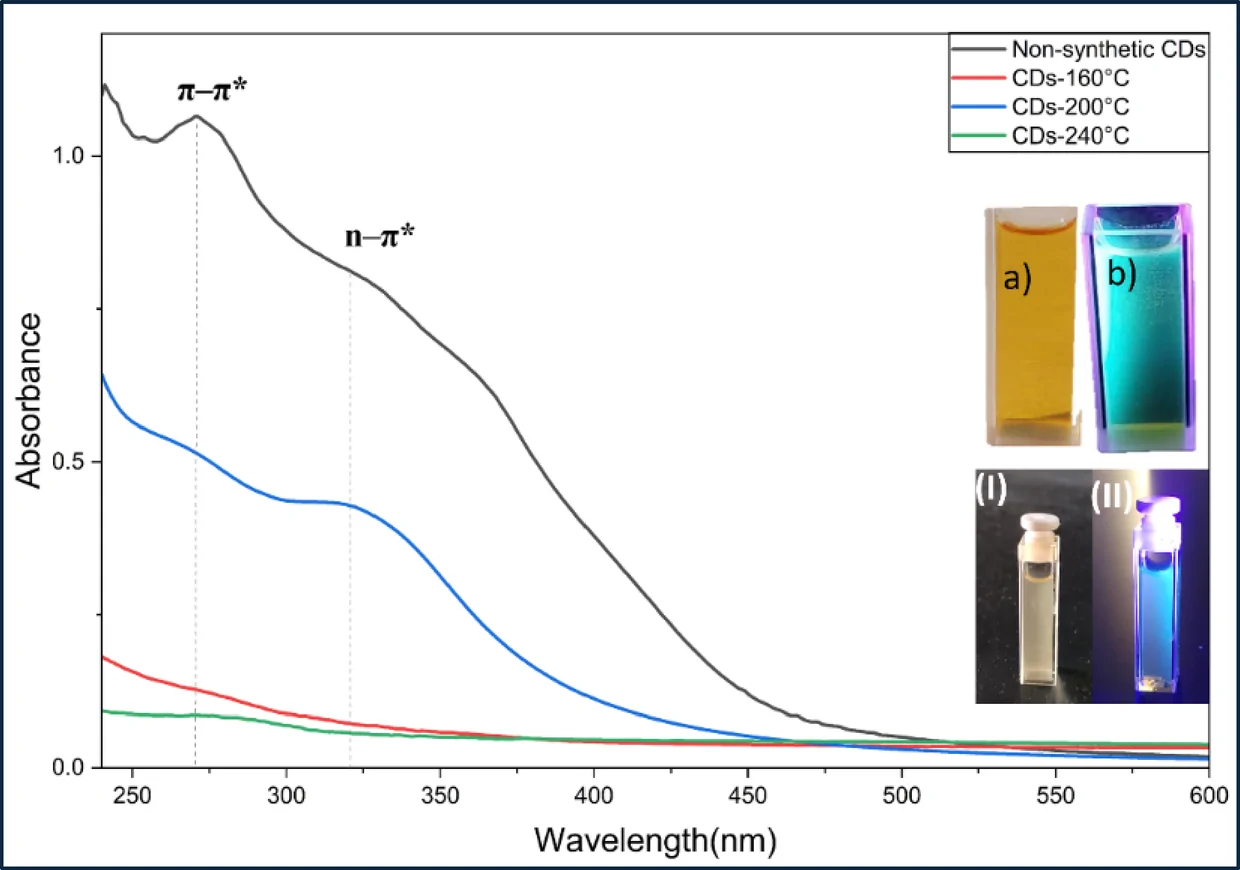
Absorbance spectrum of non-synthesized CDs and treated CDs at different temperatures.

The optical properties, such as UV-vis absorption and photoluminescence, of CS-CDs were investigated. The UV-Vis absorption spectroscopy of CDs in an aqueous solution is illustrated in Fig. 11. The inset in Fig. 11 displays the optical image of the CD aqueous solution under daylight (I) and when illuminated by a 395 nm UV lamp (II) for non-synthetic CDs and the inset (a) and (b) illustrate optical image for synthetic CDs, visible to the naked eye. The UV-Vis absorption spectra for both the non-synthetic CDs and the synthesized Carbon Dots (CDs) revealed two distinct characteristic peaks. The absorption bands at 271 nm correspond to π–π* transition of C-C/C = C bonds (aromatic ring). Additionally, the observed absorption band at 320 nm is attributed to the n–π* transition of the C = O bond (related to the sp³-hybridized carbon defects). Notably, these peaks are significantly more pronounced and well-defined in the non-synthetic sample and the CDs prepared at 200 °C. The superior spectral resolution in these two samples suggests they possess the most refined electronic structures, aligning closely with the typical optical signature of high-quality carbon dots. In contrast, the absorption features for the samples prepared at 160 °C and 240 °C appear poorly defined and relatively featureless. For the CDs-160 °C sample, this lack of clarity is attributed to incomplete carbonization, in which the corn silk precursors have not yet fully transitioned to a structured carbon core. Regarding the CDs-240 °C sample, the deterioration of the spectral shape is likely due to excessive thermal treatment, leading to particle aggregation and surface degradation. Thus, the sharp, clear spectral profile of the CDs-200 °C identifies it as the optimal synthesized material.

**Fig. 12:**
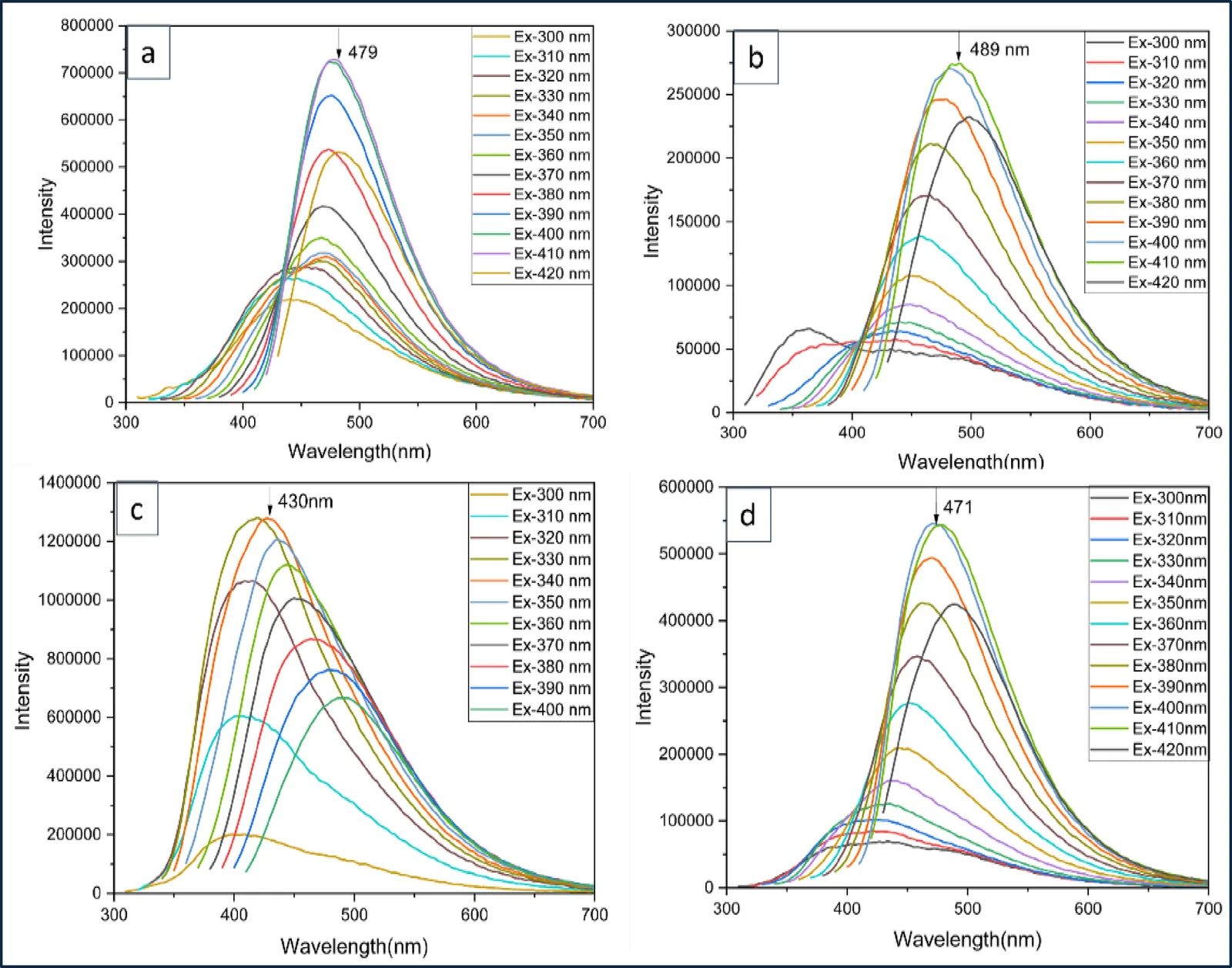
PL spectrum of the obtained (**a**) non-synthetic CDs, (**b**) CDs-160 °C, (**c**) CDs-200 °C and (**d**) CDs-240 °C.

Moreover, all CDs demonstrated excitation-dependent down-conversion photoluminescence (PL) emission^61^ (Fig. 12a–d). Namely, as excitation wavelength increased, the emitted light shifted towards longer wavelengths with a gradual decline in intensity. The CDs emitted the maximum peak intensity at 479, 489, 430, and 471 nm when excited by wavelengths 400, 410, 340, and 400 nm for non-synthetic CDs and CD samples prepared at 160, 200, 240 °C. Non-synthetic CDs showed emission at longer wavelengths; this low-energy emission from the raw material is highly likely due to native molecular fluorophores and/or random, low-energy surface defects from the precursor, rather than an optimized sp^2^ core. In contrast, CDs-200 °C showed the smallest wavelength (blue shift), 430 nm, corresponding to a high-energy electronic transition requiring a wide band gap. The hydrothermal process at 200 °C passivates the surface by converting the inefficient native defects while simultaneously creating a new set of highly uniform, highly radiative surface functional groups, which maximizes PL efficiency. CDs-160 °C illustrated that this temperature was not sufficient to carbonize or form the optimal passivating species fully. The resultant poor surface passivation leads to a high density of low-energy traps, which explains the longest emission wavelength, 489 nm, despite the expected small core size. Finally, CDs-240 °C demonstrated a shift from 430 nm to 471 nm. The higher temperature leads to more carbonization and the fusion of small carbon units, forming larger conjugated sp^2^ domains. According to the quantum confinement effect^62^, as the size of the conjugated core increases, the band gap decreases (resulting in a longer wavelength). The CDs-200 °C sample is identified as the optimum material for photoluminescence (PL) applications. Its maximum emission at 430 nm represents a sharp blue shift (high energy), signifying the most refined structure. This high-energy PL is attributed to due to optimal surface-state mechanism achieved at 200 °C. This temperature is critical because it successfully removes the inefficient, low-energy molecular fluorophores and defects present in the raw material (479 nm range) while simultaneously driving the formation of a highly uniform, highly radiative set of surface functional groups. This process creates high-energy, discrete electronic traps and effectively suppresses non-radiative decay, resulting in the widest band gap and maximized PL efficiency (quantum yield)^54^. In contrast, 160 °C yielded inefficient (red-shifted) PL due to poor passivation, while 240 °C caused over-carbonization and core fusion, leading to a detrimental (red-shift) (Quantum confinement effect) and lower efficiency^63^. The batch-to-batch reproducibility of carbon dots is a well-recognized issue in the field, and PL measurements are widely used to assess the consistency of their optical properties^64^. Photoluminescence (PL) measurements were performed on three independently prepared batches of both the non-synthetic CDs (see Fig. S4a) and the CDs synthesized at 200 °C (see Fig. S4b). As shown in Fig. S4, the emission spectra of the three batches exhibit nearly identical peak positions at 479 nm and 430 nm, respectively, when excited at 400 nm and 340 nm, along with consistent fluorescence intensities. The relative standard deviation (RSD) of the intensity was found to be 0.4% and 0.3% for the non-synthetic CDs and CDs-200 °C, respectively, confirming the excellent batch-to-batch reproducibility of the proposed green synthesis method.

### Detection of copper ions in aqueous solution

#### Selectivity of non-synthetic CDs towards metal ions

**Fig. 13:**
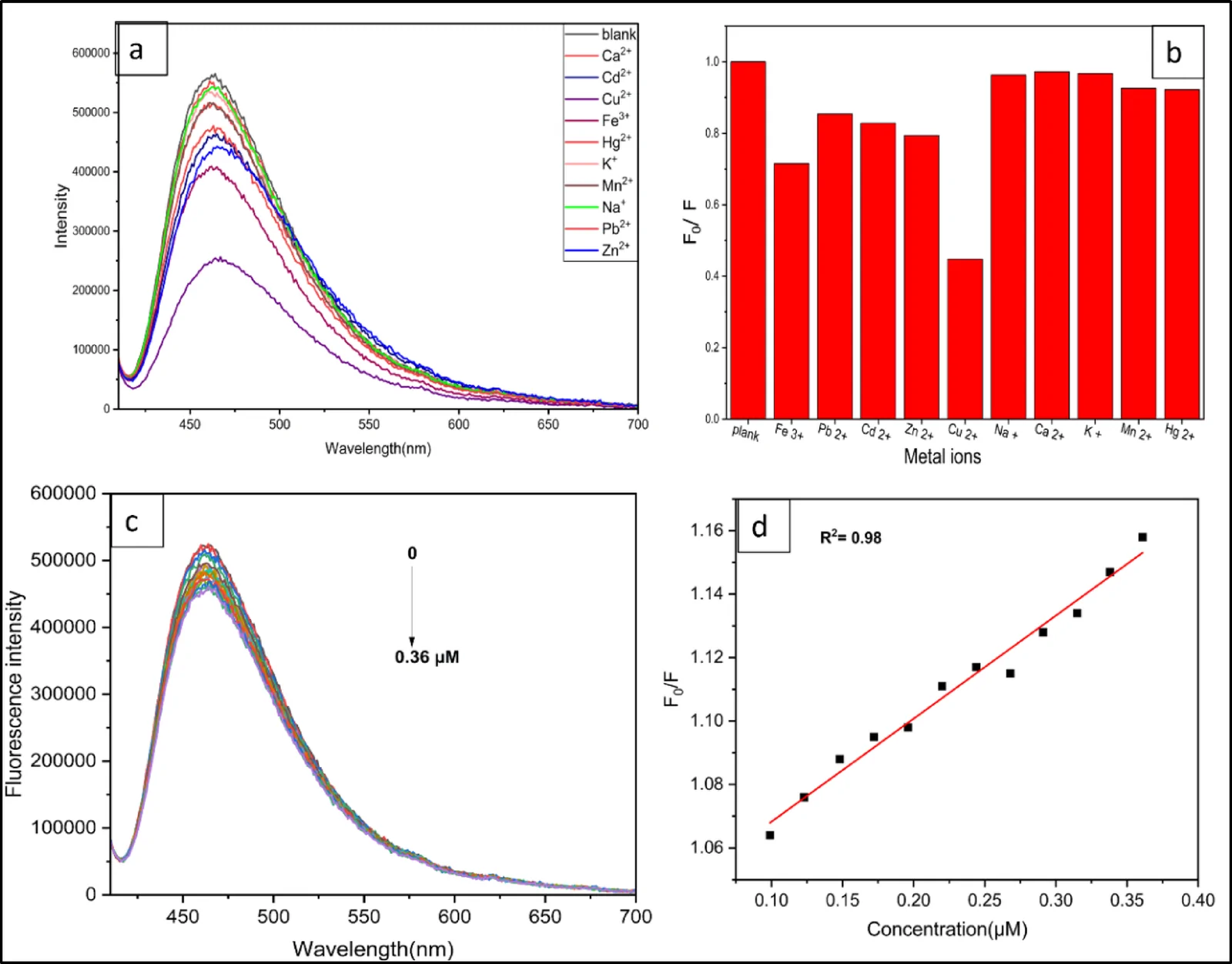
(**a**) Fluorescence response and (**b**) the histogram of non- synthetic CDs at presence of 500 µM different metal ions. (**c**) Fluorescence emission intensity quenching of non-synthetic CDs upon the addition of varying quantities of Cu^2+^ ions at excitation emission wavelengths of 400/470 nm and (**d**) the standard curve between F_0_/F and Cu^2+^ concentration).

The utilization of non-synthetic CDs as fluorescence sensors for metal ion detection has been investigated, particularly the interactions between metal ions and surface functional groups of CDs that lead to complex formation. The fluorescence emission intensity of non-synthetic CDs in the presence of several metal ions, including Ca^2+^, Cd^2+^, Cu^2+^, Fe^3+^, Hg^2+^, K^+^, Mn^2+^, Na^+^, Pb^2+^, and Zn^2+^, was assessed to evaluate their selectivity for metal ion sensing. The alterations in the fluorescence emission intensity (F/F_0_) at 470 nm (λ _ex_ ∼ 400 nm) were observed with the representative metal ions under identical conditions at a concentration of 500 µM following a 2-minute incubation period. The fluorescence intensity of carbon dots (CDs) with and without metal ions is denoted by the symbols F and F_0_, respectively (see Fig. 13a). The fluorescent behavior of carbon dots was affected differently by the metal ions. The histogram was constructed by plotting F/F_0_ in relation to the corresponding concentration of metallic ions (Fig. 13b). The sensing capability was assessed by observing the initial fluorescence intensity of CDs for notable alterations upon the addition of various metal ions. Significantly, only Cu^2+^ ions induced a pronounced and substantial quenching of fluorescence intensity in comparison to the other metal ions. When 500 µM Cu^2+^ ions were added, the fluorescence intensity decreased by up to 55%, while other metal ions showed little to no change in fluorescence intensity, leading to negligible effects. For Fe^3+^ ions, a slight quenching of about 28% was observed; this impact was not likely due to possible CD/Fe^3+^ complex formation. This outcome unequivocally demonstrates that non-synthetic CDs may serve as a highly efficient, and selective fluorescence sensor for Cu^2+^ ions owing to their enhanced affinity. The selectivity of the CDs toward Cu²⁺ in the presence of competing ions was further confirmed (Fig. S10, Supplementary Information).

#### Sensitivity of non-synthetic CDs toward Cu^2+^ Ions

For sensitivity study, various concentrations of Cu^2+^ in the range of 0 to 0.36 µM were investigated. Figure 13c shows the steady quenching of C-dots PL intensity at excitation emission wavelengths of 400/470 nm by increasing the concentration of Cu^2+^. It indicates that the sensing system is very sensitive to the Cu^2+^ concentrations. The quenching efficiencies are evaluated by the Stern−Volmer plot utilizing the subsequent Eq. (2).

2 $$\:\raisebox{1ex}{${I}_{0}$}\!\left/\:\!\raisebox{-1ex}{$I$}\right.={K}_{SV}\left(Q\right)+1$$

where I_0_ and I represent the quantum dots’ fluorescence intensity with and without Cu^2+^, [Q] is the concentration of the Cu^2+^ ion, and K_sv_ is the quenching constant. From Fig. 13d, The Stern–Volmer plot exhibited a linear relationship over the concentration range of 0–0.36 µM, with a correlation coefficient (R²) of 0.98. The limit of detection (LOD) was calculated using the equation LOD = 3σ/S, where the slope (S) was 0.3294 µM^− 1^ and the standard deviation of the intercept (σ) was 0.00359. Based on these values, the LOD was found to be 33 nM. The limit of quantitation (LOQ), calculated as 10σ/S, was determined to be 0.11 µM. In comparing the CDs in this work to past studies, it is evident that the sensing performance characteristics were comparable to those of previously published CDs (see Table 2). In addition, to assess the stability of non-synthetic CDs, the photostability test was evaluated under continuous excitation at 400 nm for 240 min with a 15-minute step, using a spectrofluorometer equipped with a xenon lamp (150 W). As shown in Fig. S5, the fluorescence intensity of the CDs remained nearly unchanged over 240 min, indicating excellent resistance to photobleaching.

### Detection of Fe^3+^ in aqueous solution

#### Selectivity of CDs-200 ͦ C towards metal ions

**Fig. 14:**
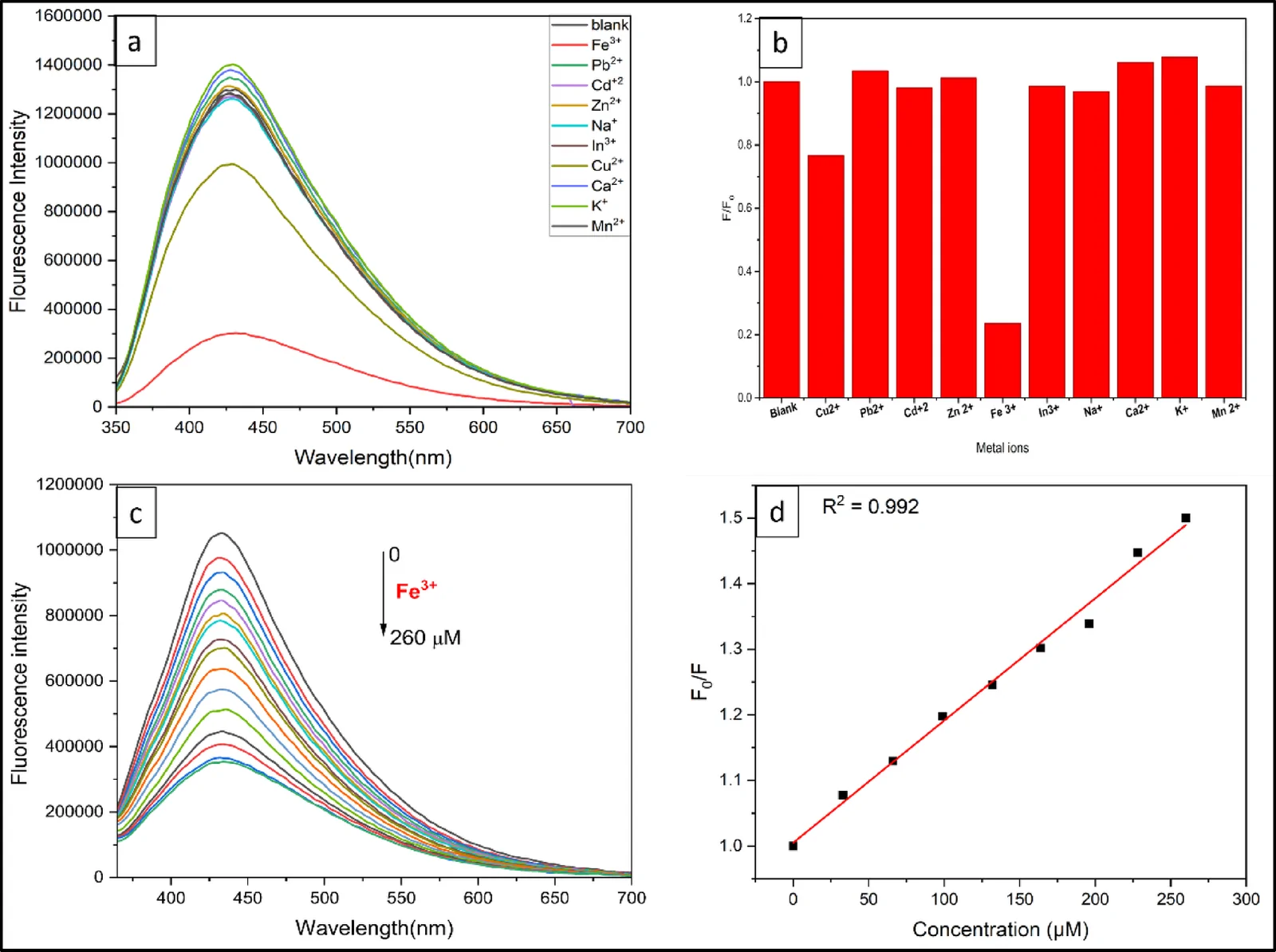
PL spectra of CDs-200 °C (**a**) upon interaction with various metal ions. (**b**) The percentage variation in fluorescence intensity of CDs-200 °C in the presence of diverse metal ions. (**c**) Fluorescence emission intensity quenching of CDs-200 °C upon the addition of varying quantities of Fe^3+^ ions at excitation emission wavelengths of 340/430 nm and (**d**) Stern Volmer plot shows the linear correlation between F_0_/F and Fe^3+^ concentration.

The use of carbon dots (CDs-200 °C) as fluorescence sensors for metal ion detection has been investigated, focusing on how metal ions interact with CD surface functional groups that can form complexes. The fluorescence emission intensity of carbon dots (CDs-200 °C) in the presence of several metal ions, including Fe^3+^, Pb^2+^, Cd^2+^, Zn^2+^, Na+, In^3+^, Cu^2+^, Ca2+, K^+^, and Mn^2+^, was assessed to evaluate their selectivity for metal ion sensing. The alterations in the fluorescence emission intensity (F/F_0_) at 430 nm (λ_ex_ ∼ 340 nm) were observed with the representative metal ions under identical conditions at a concentration of 10 mM following a 2-minute incubation period. The fluorescence intensity of carbon dots (CDs) with and without metal ions is denoted by the symbols F and F_0_, respectively (Fig. 14a). The fluorescent behavior of carbon dots was affected differently by the metal ions. The histogram was constructed using F/F_0_ in relation to the corresponding concentration of metal ions (Fig. 14b). The sensing capability was assessed by observing the initial fluorescence intensity of CDs for notable alterations upon the addition of various metal ions. Significantly, only Fe^3+^ ions induced a pronounced and substantial quenching of fluorescence intensity in comparison to the other metal ions. When 10 mM Fe^3+^ ions were added, the fluorescence intensity decreased by up to 76%, while other metal ions showed little to no change in fluorescence intensity, leading to negligible effects. For Cu^2+^ ions, a slight quenching of about 23% was observed; this impact was not notable and could be related to possible CD/Cu^2+^ complex formation. This outcome unequivocally demonstrates that CDs-200 °C may serve as a highly specific, efficient, and selective fluorescence sensor for Fe^3+^ ions owing to their enhanced affinity. The selectivity of the CDs-200 °C toward Fe^3+^ in the presence of competing ions was further confirmed (Fig. S11), Supplementary Information).

#### Sensitivity of CDs-200 °C toward Fe^3+^ ions

To evaluate the sensitivity for the quantitative detection of Fe³⁺ ions, a CDs-200 °C solution was analyzed with varying concentrations of Fe³⁺ (0–260 µM). The CDs-200 °C, in the absence of Fe³⁺, exhibited robust fluorescence emission at 430 nm (λ_ex_ = 340 nm). Upon increasing the Fe³⁺ concentration, the intensity decreased significantly, reaching a quenching efficiency of 66% at the highest concentration, while the peak position remained unchanged (Fig. 14c). To further evaluate the effect of synthesis conditions on sensing performance, CDs prepared at 160 °C and 240 °C were also examined under identical conditions. These samples exhibited lower quenching efficiencies (62.6% and 56.7%, respectively) compared to CDs-200 °C, indicating reduced sensitivity toward Fe³⁺ ions (Fig. S6). This result confirms that 200 °C provides optimal surface functionalization for enhanced Fe³⁺ detection. The Stern–Volmer plots exhibited good linearity in the range of 0–260 µM with a Pearson correlation coefficient (R² = 0.99) (Fig. 14d). The limit of detection (LOD) was calculated using the equation LOD = 3σ/S, where S is the slope of the calibration curve at low concentration and σ is the standard deviation of the y-intercept. The slope (S) was 0.00186 µM⁻¹, while σ was 0.00967. Based on these values, the LOD was determined to be 15.59 µM, whereas the limit of quantification (LOQ = 10σ/S) was 51.99 µM. In addition, the Stern–Volmer quenching constant (KSV) was calculated to be 1.86 × 10³ M⁻¹.These results are more comparable with those reported in other studies (see Table 2).

#### pH effect on sensing

pH dependence of the CDs and the CDs+ Cu^2+^ system was investigated. As shown in Fig. S2, the influence of pH on the PL intensity was significant. The PL intensity of the CDs increased with increasing pH, reaching its maximum at pH 8.0 (see Fig. S2a), followed by a decrease in basic media. This behavior is likely due to the protonation and deprotonation of carboxyl and other surface functional groups^45^. In contrast, the fluorescence quenching efficiency toward Cu²⁺ (F₀/F) was maximized at pH 7.0 (Fig. S2b). CDs fluorescence peaked at pH 8 (complete -COO⁻ and partial -NH_3_^+^ deprotonation), while Cu^2+^ quenching (F_0_/F) maximized at pH 7 (see Fig. S2b) due to optimal zwitterionic surface: deprotonated carboxylates (-COO⁻), protonated amines (-NH_3_^+^), and N-doped sites (pyridinic/pyrrolic N) enabling strong Cu^2+^ coordination without proton competition or hydroxide interference^65^.

#### The effect of interaction time

The effect of interaction time on the fluorescence quenching of CDs was investigated at two Cu²⁺ concentrations (0.48 µM and 1.0 µM). As shown in Fig. S3, a sharp decrease in fluorescence intensity was observed immediately after the addition of Cu²⁺ (t = 0 min), indicating a rapid interaction between CDs and Cu²⁺ ions. Subsequently, the fluorescence signal remained nearly constant over the entire monitored time range, demonstrating a fast response and excellent temporal stability of the sensing system^65^.

#### Discussion on the selectivity of Cu^2+^ and Fe^3+^ ions

This study investigates the similarities and differences between two carbon dots capable of detecting Cu^2+^ and Fe^3+^ ions. The comparison of non-synthetic CDs and CDs-200 °C indicated that the ratio of nitrogen to oxygen atoms may contribute to the detection of Cu^2+^ and Fe^3+^ ions. Carbon dots containing a higher concentration of N-based groups and N atoms exhibited a greater propensity for the selective detection of Cu^2+^ ions^66,67^. The higher N content in the non-synthetic CDs promotes specific Cu^2+^ binding (a borderline acid-base match), while the dominant O content in CDs-200 °C promotes Fe^3+^ binding (a hard acid-base match), which is chemically sound and provides a robust rationale for the observed selectivity. The ratio of N in non-synthetic CDs and CDs-200 °C was 5.03% and 0.28%, respectively. The Hard and Soft Acids and Bases (HSAB) theory could explain the difference: acids and bases can be defined as hard or soft. Hard refers to particles with a higher charge density and smaller radius, while soft refers to particles with a lower charge density and larger radius. The interactions between a soft acid and a soft base are more stable, while the interactions between a hard acid and a hard base are more stable. Thus, Cu^2+^ ions are borderline acids and can more easily interact with N atoms with a lower electronegativity and bigger radius; Fe^3+^ ions are similar to hard acids, so they are more likely to interact with O atoms with a higher electronegativity and smaller radius^66^.

**Table 2:** Comparison of analytical performance of different CDs for Fe ^3+^ and Cu^2+^ sensing.

Ion detected	Source	Methodology	Size (nm)	PLQY %	Analytical performance		Ref.
					Linear range (µM)	LOD (µM)	
Fe^3+^	Citric acid	Hydrothermal	1.5–4	59	20–200	20	[68]
	Poplar wood	Hydrothermal	5.02	42	0.1–100	4.1	[69]
	Coconut coir	Hydrothermal	5.64 to 10	45	(50–1) *10^3^	223.2	[70]
	Cranberry beans	Hydrothermal	3.96	10.85	30–600	9.55	[71]
	Hamburger sandwich leftover	Hydrothermal	–	–	12.5–100	32	[72]
	Vitamin B1	Hydrothermal	3.2	4.4	100–1000	0.177	[73]
	Corn silk	Hydrothermal	9	11.25	0 − 260	15.59	This Work
Cu^2+^	Rice stock	Stirrer assisted	5.4	32.61	0–260	0.032	[74]
	Citric acid	mesoporous silica (MS) spheres	3–4	∼7	0–10	0.023	[75]
	Glucose	Hydrothermal	5	–	0.01–1.00	0.007	[76]
	Grape juice	Thermolysis	8	32.1	0.8–95	0.24	[77]
	Corn straw	Hydrothermal	5	4.64	15.7- 314.7 and 314.7–7.8*10^3^	67.04	[78]
	Corn silk	Non-synthetic	6.7	1.86	0-0.36	0.033	This work

#### Identification of Fe^3+^ in actual water samples

**Fig. 15:**
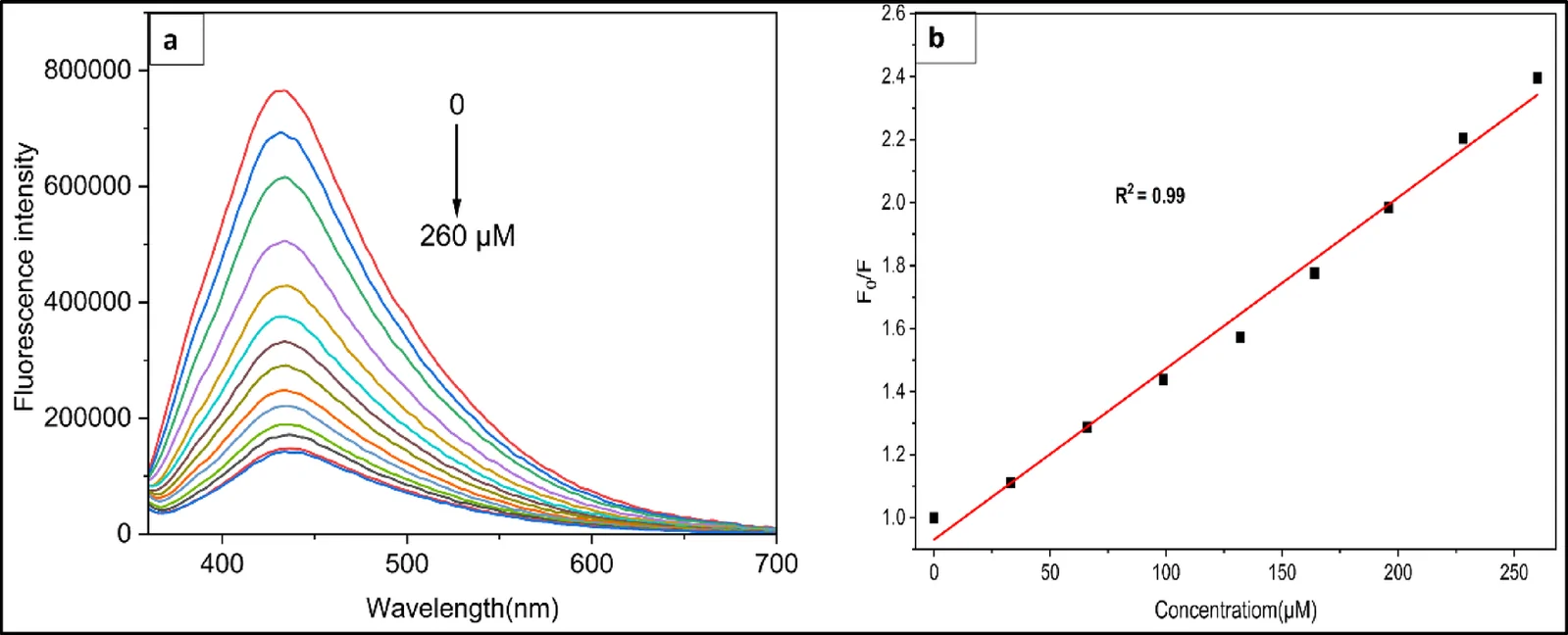
(**a**) Fluorescence emission spectra of CDs in the presence of different concentration of Fe^3+^ in tap water and (**b**) The plot of F_0_/F versus the concentration of Fe^3+^ in tap water (displays the linear relationship between F_0_/F and Fe^3+^ concentration).

To assess the feasibility of the fluorescent probe for practical applications, a standard addition (spiking) method was employed using tap water collected from our laboratory and bottled water. Bottled water was additionally analyzed to further validate the applicability of the proposed method. Both samples were filtered through a 0.22 μm syringe filter prior to use. Different concentrations of Fe³⁺ were added to the CDs solution, resulting in a gradual decrease in fluorescence intensity for both tap water (see Fig. 15a) and bottled water (see Fig. S9a). The Stern–Volmer plots exhibited good linearity (Fig. 15b). A linear relationship was observed in the range of 0–260 µM for tap water (R² = 0.99), consistent with that obtained for CDs alone, while in bottled water, linearity was observed in the range of 0–148 µM (R² = 0.99) (see Fig. S9b). To further evaluate the analytical performance, known concentrations of Fe³⁺ (66, 99, and 196 µM) were spiked into tap water, while 118.58, 99.01, and 141.3 µM were added to bottled water. The recovery values ranged from 99.05% to 104.16%, indicating good accuracy of the proposed method. The relative standard deviation (RSD%) values were low, demonstrating good precision of the sensor. The detailed results are summarized in Table 3.

**Table 3:** Recovery results and spiking recoveries of Fe^3+^ in Tap water (*n* = 3).

Sample	Spike d Fe^3+^ (µM)	Found Fe^3+^(µM)	Recovery (%)	Relative standard deviation (%) (*n* = 3)
Tap water	196	196.98 ± 2.78	100.5 ± 1.42	1.411
	99	98.06 ± 2.73	99.05 ± 2.76	2.78
	66	68.76 ± 4.56	104.16 ± 6.9	6.16
Bottle-water	118.58	119.3 ± 0.89	100.6 ± 0.75	0.75
	99	102.88 ± 0.69	103.92 ± 0.69	0.67
	141.3	143.3 ± 0.92	101.41 ± 0.65	0.65

#### The time-resolved fluorescence of the non-synthetic CDs and synthetic CDs at 200 °C

To obtain a comprehensive understanding of the quenching of this system, time-resolved fluorescence was employed. The fluorescence decay characteristics of two carbon dots, both without and with metal ions (Fe^3+^ and Cu^2+^) were illustrated in Eq. (2)

3 $$\:{{\uptau\:}}_{\mathrm{A}\mathrm{v}}\:=\:\frac{{\sum\:}_{i=1}^{2}{B}_{i}\:{{{\uptau\:}}_{i}}^{2}}{{\sum\:}_{i=1}^{2}{B}_{i}\:{{{\uptau\:}}_{i}}^{2}}=\frac{{B}_{1}{{{\uptau\:}}_{1}}^{2}+\:{B}_{2}{{{\uptau\:}}_{2}}^{2}}{{B}_{1}{{\uptau\:}}_{1\:}+\:{B}_{2}{{\uptau\:}}_{2}}$$

**Fig. 16:**
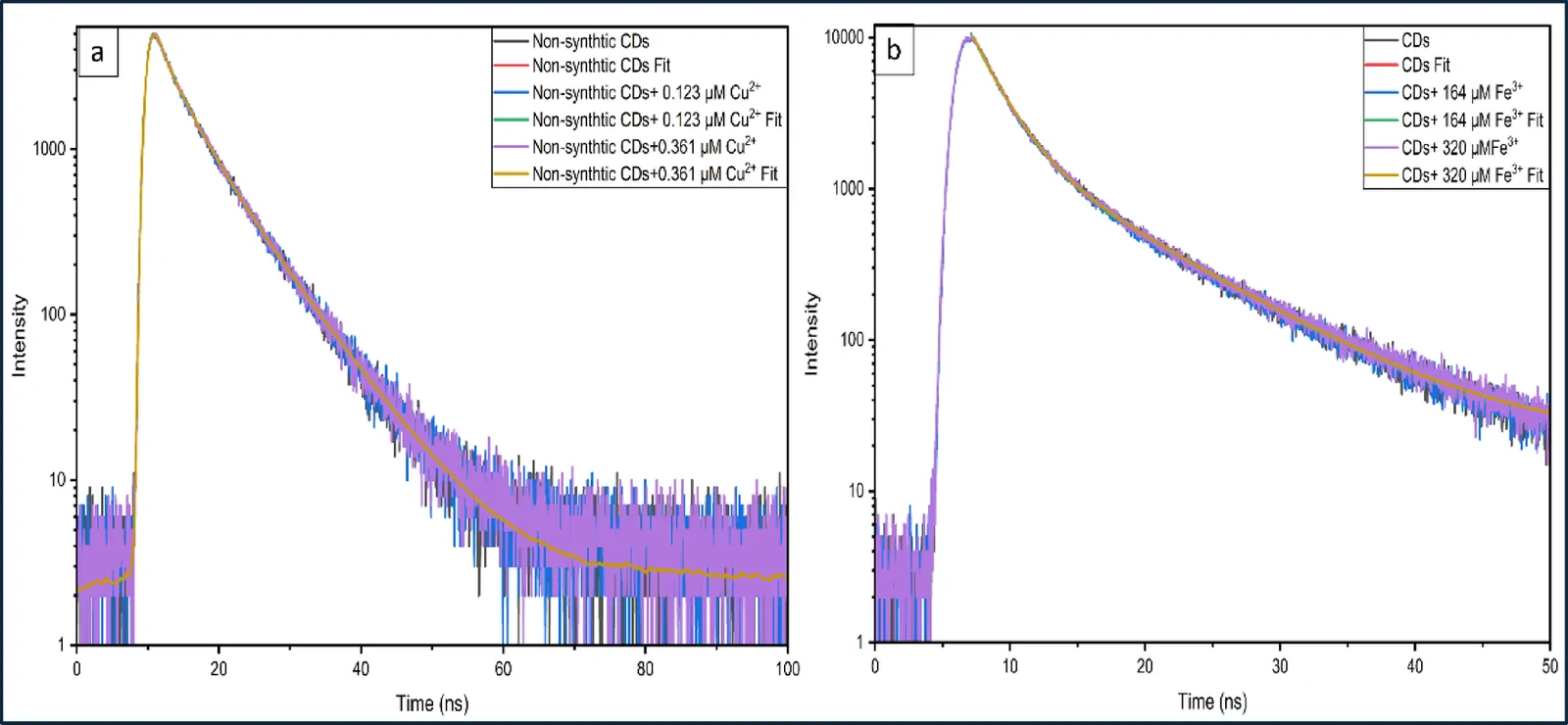
The time-resolved photoluminescence decay curve of the non-synthetic CDs alone and with different concentration of Cu^2+^ (**a**) and CDs prepared at 200 °C with different concentrations of Fe^3+^ (**b**).

Non-synthetic CDs exhibited a tri-exponential decay curve due to the highest degree of heterogeneity (see Fig. 16a). The non-synthetic CDs consist of a complex mixture containing uncarbonized molecular fluorophores and a polydisperse distribution of surface groups (including a high N content), as confirmed by XPS analysis. Therefore, non-synthetic CDs exhibited three exponential decays, assigned to unreacted molecular precursors or small, highly localized surface defect sites (short lifetime 0.691 ns), surface functional groups (medium lifetime 3.2 ns), and sp² hybridized core domains (long lifetime 7.266 ns). While the shift to a bi-exponential decay curve in CDs-200 °C indicates a reduction in heterogeneity, with a very low N content of 0.28%, the surface is dominated by fewer types of O-groups, such as C = O, and a more uniform population of sp² core due to the controlled, high-temperature synthesis. As a result, CDs-200 °C showed bi-exponential decay (see Fig. 16b) attributed to the O-rich (short lifetime 2.075 ns) surface state and the sp² core state (long lifetime 8.342 ns)^79^ as shown in Table 4. The fluorescence quantum yield (FLQY) of non-synthetic CDs was 1.86%, which was relatively higher than most CDs extracted from different sources^80–83^. In contrast, CDs synthesized at 200 °C showed 11.25%.

**Table 4:** Fluorescence lifetimes for non-synthetic CDs and CDs synthesized at 200 °C alone and with different concentrations of Cu^2+^ and Fe^3+^, respectively.

Sample		Different lifetimes (ns)	Amplitudes	Average lifetime (ns)
Non-synthetic CDs	Without Cu^2+^	τ_1_ = 0.691	B_1_=0.026	τ_Av_ = 5.33
		τ_2_ = 3.2	B_2_=0.0165	
		τ_3_ = 7.266	B_3_=0.0134	
	With 0.123 µM Cu^2+^	τ_1_ = 0.707	B_1_=0.0268	τ_Av_ = 5.32
		τ_2_ = 3.77	B_2_=0.0171	
		τ_3_ = 7.6	B_3_=0.0108	
	With 0.361 µM Cu^2+^	τ_1_ = 0.64	B_1_=0.0271	τ_Av_ = 5.38
		τ_2_ = 3.51	B_2_=0.017	
		τ_3_ = 7.53	B_3_=0.012	
CDs-200 °C	Without Fe^3+^	τ_1_ = 2.075	B_1_=8476.87	τ_Av_ = 5.2
		τ_2_ = 8.342	B_2_= 2111.56	
	With 164 µM Fe^3+^	τ_1_ = 2.095	B_1_= 8702.14	τ _Av_ = 5.16
		τ_2_ = 8.417	B_2_=2034.6	
	With 320 µM Fe^3+^	τ_1_ = 2.014	B_1_=7879.8579	τ _Av =_ 5.26
		τ_2_ = 8.182	B_2_=2163.4744	

#### Quenching mechanism

**Fig. 17:**
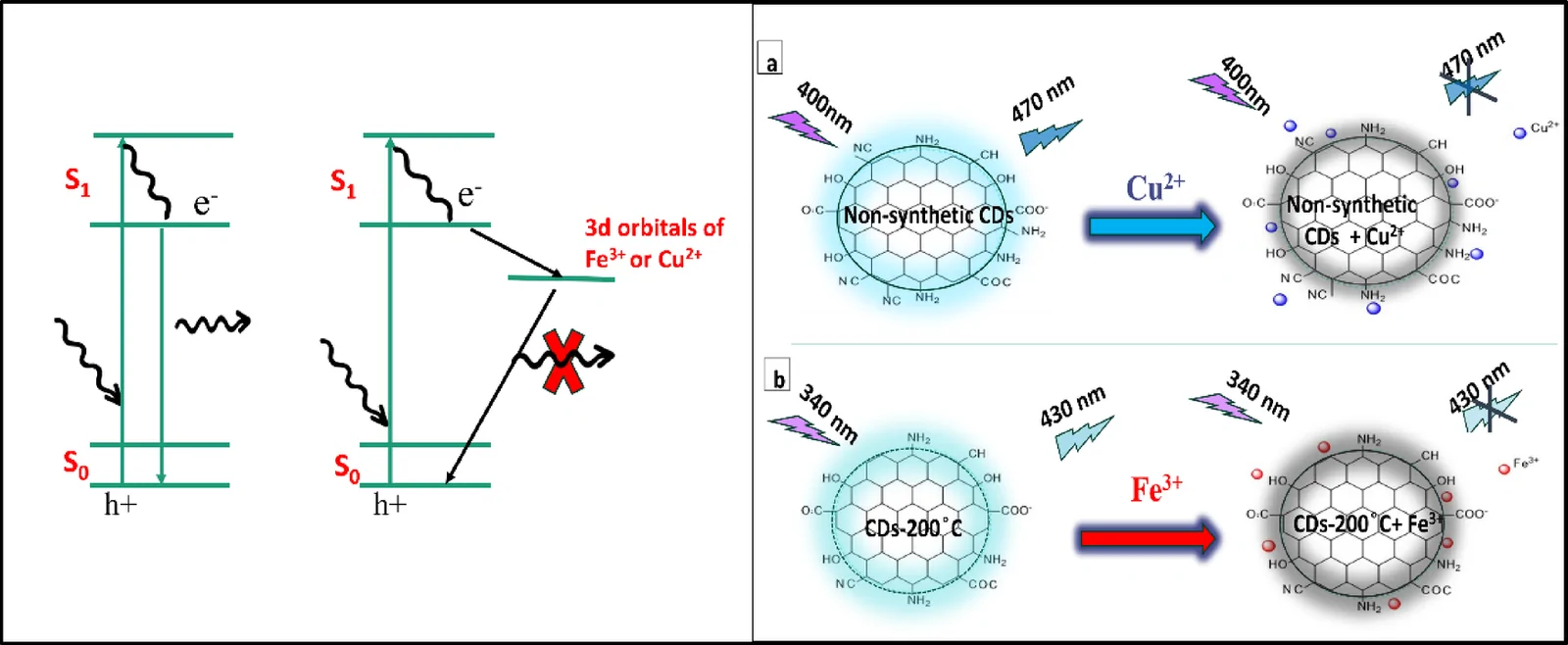
Schematic representation of chelation quenched fluorescence in detection of Cu^2+^ (**a**) and (**b**) Fe^3+^ ions.

From Table 4, the average fluorescence lifetime of the non-synthetic CDs remains essentially unchanged (around 5.3 ns regardless of the presence and concentration of Cu^2+^). Similarly, the average lifetime (τ _Av_) of the CDs-200 °C also remains unchanged (around 5.2 ns) despite the presence and increasing concentration of Fe^3+^. Since the fluorescence lifetime (τ _Av_) is not affected by the addition of quenchers (Cu^2+^ and Fe^3+^), this highlights a static quenching mechanism. Within static quenching, the quencher (Cu^2+^ and Fe^3+^) and fluorophore (the CDs) form a stable, non-fluorescent complex (a ground-state complex) before excitation. This complex formation reduces the concentration of the fluorophore, leading to a decrease in intensity without altering the fluorescence lifetime (Fig. 17)^84^.

### Biological properties

#### Antimicrobial activity of CD samples

**Fig. 18:**
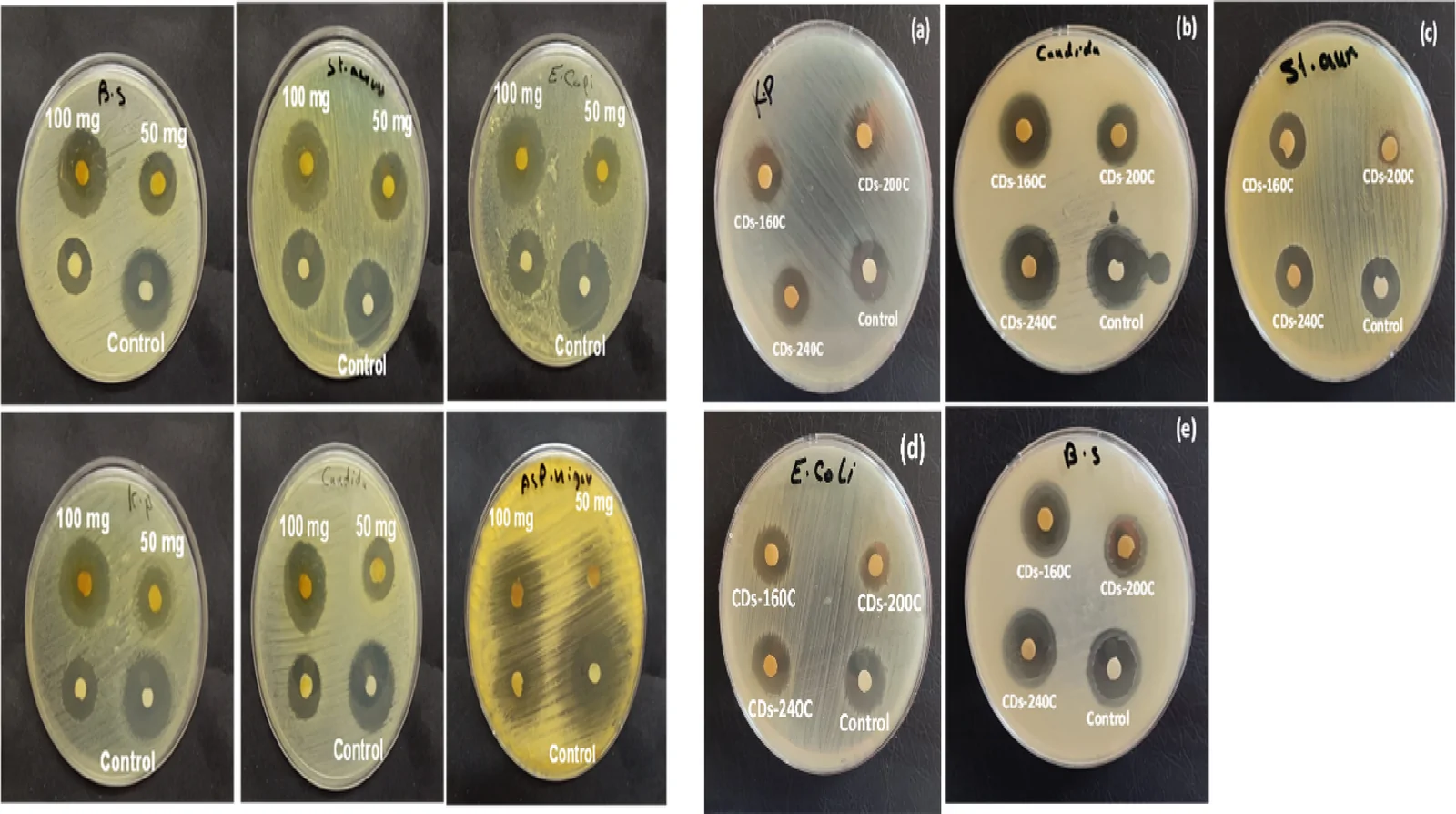
(**a**) Six images illustrating the antimicrobial activity of non-synthetic CDs at different concentrations, and (**b**) five images of culture dishes demonstrating the antimicrobial activity of CDs synthesized at 200 °C.

Comparing the three synthesized CDs-160 °C, CDs-200 °C, CDs-240 °C in the minimum inhibition concentration (MIC) values in Table 6, the 240 °C sample consistently demonstrates the highest potency (lowest MIC) against both Gram-negative bacteria and, most significantly, the fungal strain C. albicans. The 200 °C sample showed poor efficacy in most cases (e.g., MIC = 250 µg/mL against K. pneumoniae). The 240 °C synthesis temperature yields the best therapeutic candidate and will serve as the benchmark for comparison with the non-synthetic material. While the thermal synthesis process at 240 °C maintained the high intrinsic antibacterial potency of the non-synthetic CDs evidenced by the identical MIC values of 15.62 µg/mL against *E. coli* and equivalent values for other bacterial strains it resulted in a significant enhancement of antifungal efficacy. The 240 °C synthesized CD achieved an MIC of 15.62 µg/mL against *C. albicans*, which is an eight-fold improvement in potency compared to the non-synthetic CD (MIC = 125 µg/mL). Higher synthesis temperatures promote amino group decomposition, exposing surface traps that enhance reactive oxygen species (ROS) generation via photoexcited electron transfer^85,86^. The formation of these new C = O groups drives an 8-fold enhancement in antifungal potency against *C. albicans*. The Zone of Inhibition (ZOI) results corroborate the MIC data. At the 50 µg/mL concentration for non-synthetic CDs, the ZOI against *E. coli* is essentially identical (18 ± 2 mm vs. 18 ± 1 mm), aligning with the shared MIC value. Crucially, the 240 °C synthesized CD produces a larger ZOI against *C. albicans* (20 ± 1 mm vs. 15 ± 2 mm for non-synthetic CDs) as shown in Table 6, confirming its superior efficacy not only in potency MIC but also in physical diffusion in the agar against the fungal strain as shown in Fig. 18. The CDs-240 °C demonstrate a larger effect than the antibiotic on the three bacterial species. CDs-240 °C nearly match the antibiotic’s efficacy against *S. aureus* and the fungi *Candida albicans*. However, CDs at different temperatures did not show any effect on *Aspergillus niger*. The antibacterial and antifungal activity of non-synthesized CDs increased linearly with increasing concentrations (mg/mL), indicating that these CDs can effectively suppress bacterial growth in a concentration-dependent manner as shown in Tables 5, 6; Fig. 18a. Figure 18b presents photos of culture dishes subjected to three samples of synthesized CD solution alongside a commercial antibiotic as a control.

**Table 5:** Antimicrobial activity of non- synthetic CDs in various concentrations.

No. bacterial microorganism	Zone of inhibition (mm)		Control
	50 mg/mL	100 mg/mL	1 mg/mL
Gram-positive bacteria			
1. *Bacillus subtilis*	15 ± 0.2	23 ± 2	24 ± 1
2. *Staph. aureus*	17 ± 0.2	24 ± 1	25 ± 1
Gram-negative bacteria			
3. *Escherichia coli*	18 ± 2	26 ± 1	28 ± 0.2
4. *K. pneumoniae*	16 ± 1	24 ± 1	25 ± 0.1
No. fungal cells			
5. *Candida albicans*	15 ± 2	22 ± 1	24 ± 2
6. *Aspergillus niger*	15 ± 1	25 ± 1	35 ± 1

**Table 6:** Antimicrobial activity of non-synthetic and synthesized CDs at different temperatures and MIC values.

No. bacterial microorganism	Zone of inhibition (mm)				Control (1 mg/mL)	MIC (µg /mL)			
	Non-synthetic CDs	160 °C	200 °C	240 °C		Non-synthetic CDs	160 °C	200 °C	240 °C
Gram-positive bacteria					Gentamycin				
1. *B. subtilis*	15 ± 0.2	17 ± 1	11 ± 1	20 ± 1	19±0.01	31.25	31.25	62.5	31.25
2. *S. aureus*	17 ± 0.2	14 ± 0	8 ± 0	16 ± 2	16 ± 0.2	31.25	31.25	125	31.25
Gram-negative bacteria					Gentamycin				
3. *E. coli*	18 ± 2	15 ± 1	13 ± 0	18 ± 1	16 ± 0.1	15.62	31.25	125	15.62
4. *K. pneumoniae*	16 ± 1	14 ± 1	12 ± 1	16 ± 2	15 ± 0.1	31.25	125	250	31.25
No. Fungal cells					Fluconazole				
5. *Candida albicans*	15 ± 2	16 ± 0	15 ± 1	20 ± 1	20 ± 0.2	125	62.5	125	15.62
6. *Aspergillus niger*	15 ± 1	NA	NA	NA	32 ± 0.1	17.5	–	–	–

### Cytotoxicity and cell morphological assay

**Fig. 19:**
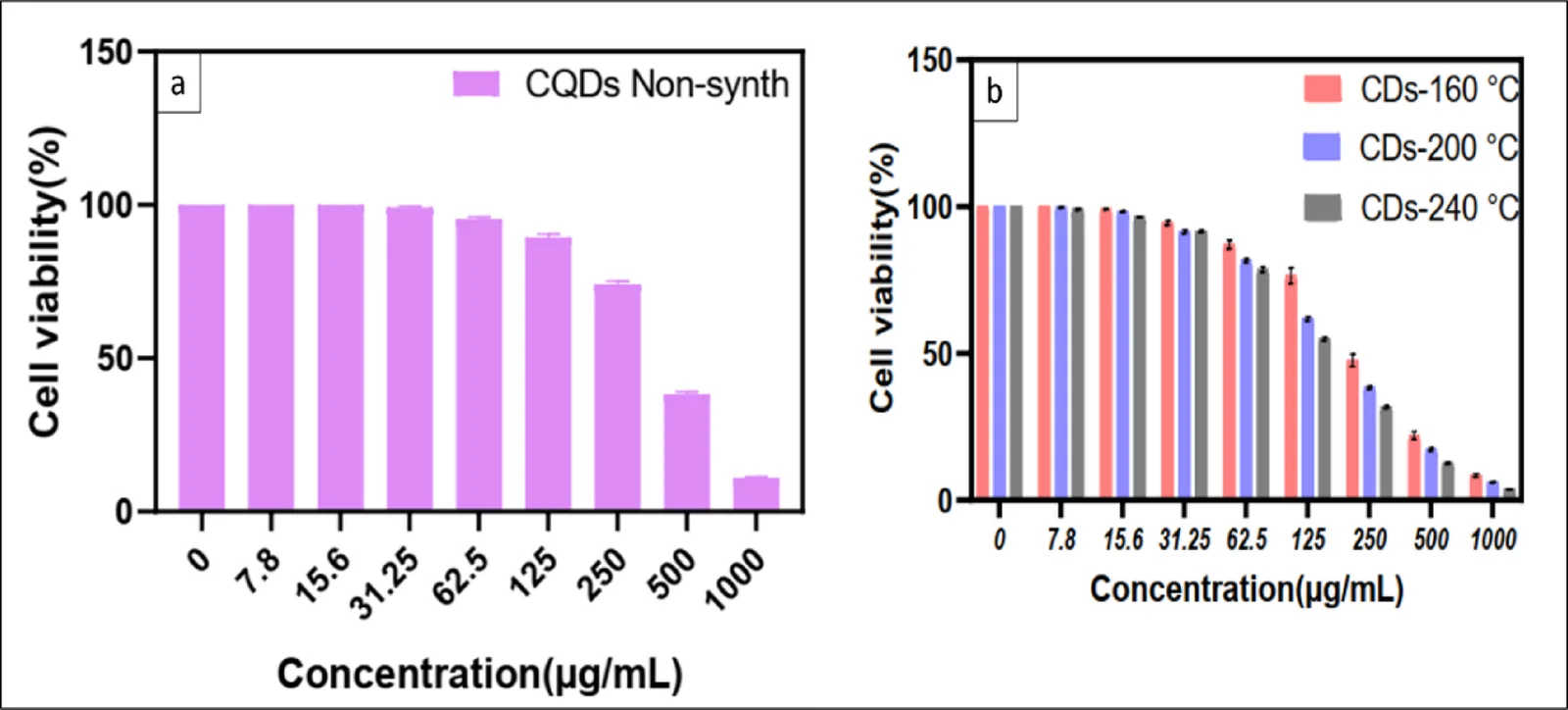
The percentage of cell viability as a function of non-synthetic CDs (**a**) and CDs prepared at 160 °C, 200 °C and 240 °C concentrations (**b**).

**Fig. 20:**
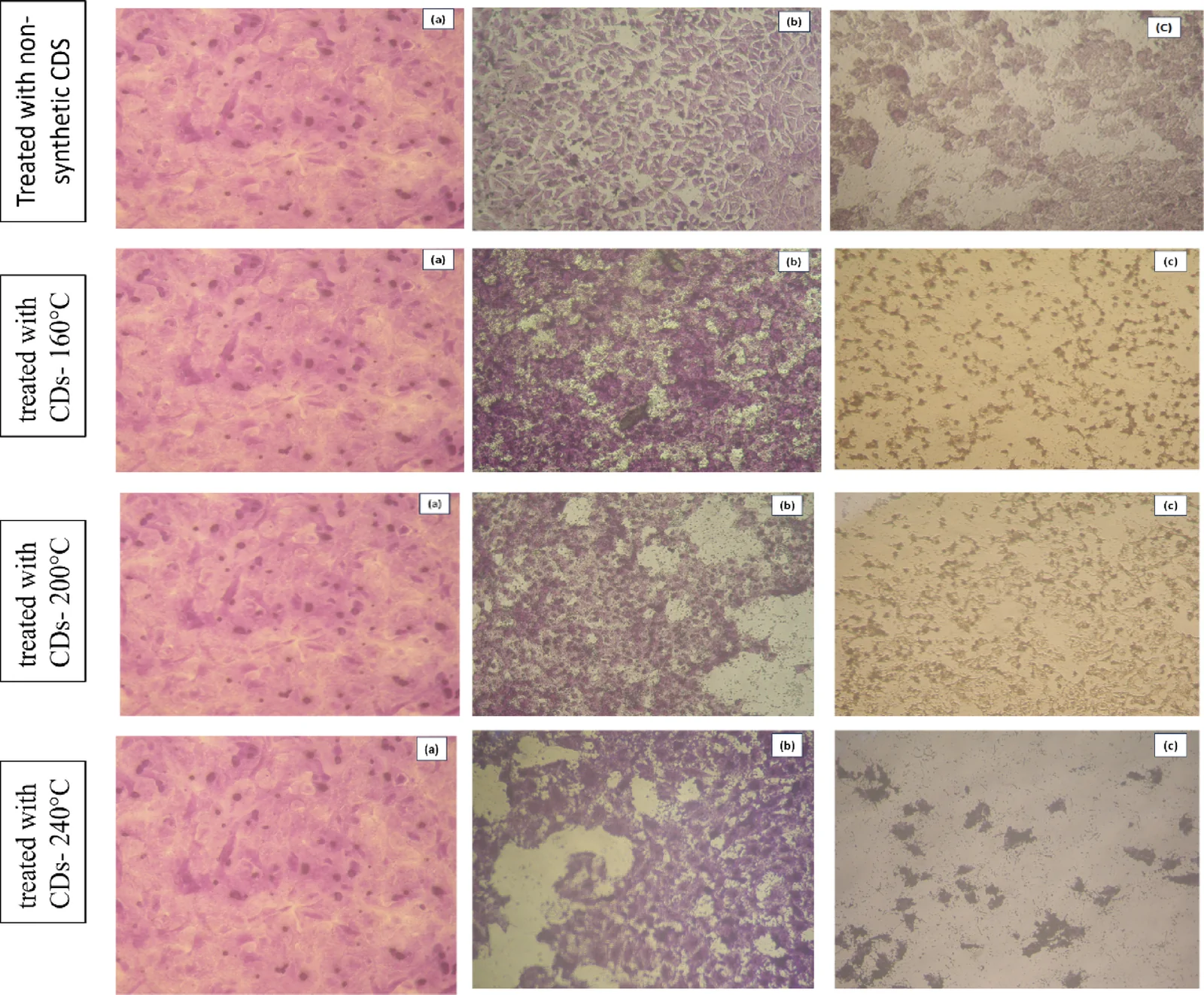
MCF7-cel lines control (**a**) treated with CD samples at concentrations 250 µg/mL (**b**) and 1000 µg/mL (**c**).

The present study aims to estimate the cytotoxicity of non-synthetic and synthetic CDs at different temperatures using MTT assay through the viabilities of Michigan Cancer Foundation-7 (MCF-7) Breast cancer cells. Figure 19a shows the cell viabilities of CDs naturally extracted from corn silk with different concentrations (7.8, 15.6, 31.25, 62.5, 125,250,500,1000 µg/mL). These non-synthetic CDs at concentrations ranging from 0 to 150 µg/mL to MCF-7 cells did not weaken the cell viability significantly, confirming that non-synthetic CDs have low cytotoxicity and could be utilized in bioimaging and other biomedical applications at high concentrations. It is significant that non-synthetic CDs exhibited a higher half-maximal inhibitory concentration (IC_50_) of 418.17 ± 6.31 µg/mL, thereby confirming their cytocompatibility. Figure 19b illustrates the dose-dependent cytotoxicity of three samples CDs-160 °C, CDs-200 °C and CDs-240 °C. As the concentrations of the additional substances escalate, cell viability diminishes systematically. The IC_50_ for the CDs-160 °C, CDs-200 °C, and CDs-240 °C are determined to be 245.46, 187.68, and 151.16 µg/mL, respectively. These values mean that there is no substantial reduction in the viability of breast cancer cells (MCF-7), even at elevated doses of CDs. All CD samples demonstrated low cytotoxicity, which supports their suitability for biomedical applications. The non-synthetic CDs recorded the highest IC_50_, indicating the greatest cytocompatibility among the tested samples. In addition, the images obtained from the inverted microscope demonstrate cell inhibition following treatment with natural extracted CQDs. The cytopathic effects, characterized by morphological alterations, were observed microscopically at a magnification of 200x, as illustrated in Fig. 20. This study illustrates the alterations in the morphological characteristics of the MCF-7 cell line following treatment with non-synthesized and synthesized CDs. MCF-7 cells are generally described as having an epithelial-like morphology^87^. This means they tend to adhere to surfaces and form monolayers. In addition, these cells are polygonal or spindle-shaped and tightly packed^88^. Therefore, when treated with CDs, a progressive shift from the cells’ normal, adherent, spindle or polygonal morphology to irregular, detached cells, with a simultaneous expansion of intercellular space. At higher concentrations, increased cellular shrinkage was observed, demonstrating a dose-dependent response. The effect was most pronounced with CDs-240 °C, consistent with previous cell viability findings.

## Conclusion

This study successfully leveraged the dual issues of agricultural waste and environmental contamination by developing novel synthesis and extraction pathways for multi-functional carbon dots (CDs) derived from corn silk. By comparing direct extraction with hydrothermal synthesis across temperatures ranging from 160 °C to 240 °C we established that the synthesis temperature is the critical factor governing the final CDs structure and its functional specialization, revealing distinct optima for therapeutic and optical applications. The 240 °C synthesis temperature was definitively identified as the therapeutic optimum. Furthermore, both the extracted and synthesized CDs proved highly effective as fluorescent sensors. The non-synthetic CDs were demonstrated to be selective for Cu^2+^ ions with an outstanding Limit of Detection (LOD) of 32 nM. This selectivity was dictated by the elemental composition, with the high Nitrogen content in the non-synthetic material promoting specific binding of Cu^2+^, while oxygen-rich synthesized CDs-200 °C favored Fe^3+^ sensing with a low detection limit of 15.59 µM. This research not only presents a versatile, sustainable platform for CD production but also delivers essential control principles. The optimized CDs-240 °C material demonstrates clinical relevance, with ZOI values matching or exceeding commercial antibiotics, marking it as a highly promising, dual-application material for advanced sensing and next-generation antimicrobial therapy.

## Supplementary Information

Below is the link to the electronic supplementary material.

## Data Availability

Data for this study are available upon request from the corresponding author.

## Abbreviations

CS: Corn silk
CDs: Carbon dots
PL: Photoluminescence
MIC: Minimum inhibitory concentration
LOD: Limit of detection
QY: Quantum yield

## References

[1] Lai, W. T. et al. A review: Modified agricultural by-products for the development and fortification of food products and nutraceuticals. *Trends Food Sci. Technol.* **59**, 148–160. 10.1016/J.TIFS.2016.11.014 (2017).

[2] *IGC Maintains Global Wheat, Corn Production Forecasts. Hellenic Shipping News Worldwide*. https://www.hellenicshippingnews.com/igc-maintains-global-wheat-corn-production-forecasts/ (n.d.).

[3] Maksimovíc, Z., Maleňcíc, D. & Kovǎcevíc, N. Polyphenol contents and antioxidant activity of Maydis stigma extracts. *Bioresour. Technol.* **96**, 873–877. 10.1016/J.BIORTECH.2004.09.006 (2005). 15627557

[4] da Hora, N. R. S. et al. Identification of bioactive metabolites from corn silk extracts by a combination of metabolite profiling, univariate statistical analysis and chemometrics. *Food Chem.* **365**, 130479. 10.1016/J.FOODCHEM.2021.130479 (2021). 34229991

[5] Asiri, S. A., Matar, A. & Ismail, A. M. Chemical composition, physical properties, and sensory evaluation of wheat-based cookies enriched with different proportions of corn silk. *Ital. J. Food Sci.* **36**, 317–330. 10.15586/IJFS.V36I4.2654 (2024).

[6] Orellana-Palacios, J. C. et al. Corn silk as an underutilized sustainable source of plant proteins: Extraction, fractionation, structure, and techno-functional properties. *Food Hydrocoll.* **158**, 550. 10.1016/j.foodhyd.2024.110550 (2025).

[7] Abdelmonsef, D., Hammad, S. & Ghali, M. Synthesis of carbon quantum dots from corn silk. *J. Nano Res.* **89**, 45–50. 10.4028/p-u75H0z (2025).

[8] Zheng, J. Q., Yan, M., Jiang, H. J. & Yin, T. P. Carbon dots derived from corn silk and their antimicrobial properties. *Food Med. Homology*. **3**, 9420094. 10.26599/FMH.2026.9420094 (2026).

[9] Archana, P., Mayil Vealan, S. B. & Sekar, C. Qualitative and quantitative detection of cyanide in forage crop and fibre dietary products by colorimetric and fluorescent sensor based on corn silk derived carbon dots-rutin system. *Food Chem.* **493**, 145978. 10.1016/J.FOODCHEM.2025.145978 (2025). 40840381

[10] Bakier, Y. M., Ghali, M. & Zahra, W. K. Highly sensitive fluorescent detection of pyridine using small size carbon quantum dots derived from folic acid. *J. Phys. D Appl. Phys.* **53**, 10. 10.1088/1361-6463/ab985e (2020).

[11] Atchudan, R. et al. Tunable fluorescent carbon dots from biowaste as fluorescence ink and imaging human normal and cancer cells. *Environ. Res.* **204**, 112365. 10.1016/J.ENVRES.2021.112365 (2022). 34767820

[12] Boobalan, T. et al. Mushroom-derived carbon dots for toxic metal ion detection and as antibacterial and anticancer agents. *ACS Appl. Nano Mater.* **3**, 5910–5919. 10.1021/ACSANM.0C01058/SUPPL_FILE/AN0C01058_SI_001.PDF (2020).

[13] Bassetti, S., Tschudin-Sutter, S., Egli, A. & Osthoff, M. Optimizing antibiotic therapies to reduce the risk of bacterial resistance. *Eur. J. Intern. Med.* **99**, 7–12. 10.1016/J.EJIM.2022.01.029 (2022). 35074246

[14] Hao, X. et al. Antibacterial activity of positively charged carbon quantum dots without detectable resistance for wound healing with mixed bacteria infection. *Mater. Sci. Eng. C*. **123**, 111971. 10.1016/J.MSEC.2021.111971 (2021). 33812599

[15] Zhou, Q. et al. Large-scale electrochemical fabrication of nitrogen-doped carbon quantum dots and their application as corrosion inhibitor for copper. *J. Mater. Sci.* **56**, 12909–12919. 10.1007/S10853-021-06102-X/FIGURES/6 (2021).

[16] Karuppasamy, B. D. et al. Hydrothermal synthesis of intrinsic carbon dots with tunable emission for fluorescent inks and variegated bioimaging applications. *J. Mol. Liq*. **437**, 128592. 10.1016/J.MOLLIQ.2025.128592 (2025).

[17] Aly, A., Ghali, M., Osman, A., El, M. K. & Nimr Non-synthetic luminescent graphene quantum dots in coconut water for aniline sensing applications. *Mater. Res. Bull.* **171**, 112603. 10.1016/J.MATERRESBULL.2023.112603 (2024).

[18] Appenroth, K. J. What are heavy metals in plant sciences? *Acta Physiol. Plant.* **32**, 615–619. 10.1007/S11738-009-0455-4/FIGURES/1 (2010).

[19] Thai, T. D., Lim, W. & Na, D. Synthetic bacteria for the detection and bioremediation of heavy metals. *Front. Bioeng. Biotechnol.* **11**, 680. 10.3389/fbioe.2023.1178680 (2023). PMC10133563 37122866

[20] Thai, T. D., Lim, W. & Na, D. Synthetic bacteria for the detection and bioremediation of heavy metals. *Front. Bioeng. Biotechnol.* **11**, 680. 10.3389/fbioe.2023.1178680 (2023). PMC10133563 37122866

[21] Ning, G., Li, B., Liu, J., Xiao, Q. & Huang, S. Red-emission carbon dots as fluorescent on–off–on probe for highly sensitive and selective detection of Cu2 + and glutathione. *Anal. Bioanal Chem.* **414**, 2219–2233. 10.1007/s00216-021-03859-7 (2022). 34988589

[22] Zong, J. et al. Carbon dots as fluorescent probes for off-on detection of Cu2 + and l-cysteine in aqueous solution. *Biosens. Bioelectron.* **51**, 330–335. 10.1016/j.bios.2013.07.042 (2014). 23994615

[23] Singh, V. K. et al. In situ functionalized fluorescent WS2-QDs as sensitive and selective probe for Fe3 + and a detailed study of its fluorescence quenching. *ACS Appl. Nano Mater.* **2**, 566–576. 10.1021/ACSANM.8B02162 (2019).

[24] Mondal, D., Datta, S. & Jana, D. Navigating the evolution of two-dimensional carbon nitride research: integrating machine learning into conventional approaches. *Phys. Chem. Chem. Phys.* **27**, 4531–4566. 10.1039/D4CP04309J (2025). 39935374

[25] Khalkho, B. R. et al. A simple and convenient dry-state SEIRS method for glutathione detection based on citrate functionalized silver nanoparticles in human biological fluids. *New J. Chem.* **45**, 1339–1354. 10.1039/d0nj04065g (2021).

[26] Zhu, X., Kalyanaraman, N. & Subramanian, R. Enhanced screening of glutathione-trapped reactive metabolites by in-source collision-induced dissociation and extraction of product ion using UHPLC-high resolution mass spectrometry. *Anal. Chem.* **83**, 9516–9523. 10.1021/ac202280f (2011). 22077671

[27] Bagheri, N. et al. Electroanalytical sensor based on gold-nanoparticle-decorated paper for sensitive detection of copper ions in sweat and serum. *Anal. Chem.* **93**, 5225–5233. 10.1021/acs.analchem.0c05469 (2021). 33739824

[28] Tsiasioti, A., Zotou, A. S. & Tzanavaras, P. D. Single run analysis of glutathione and its disulfide in food samples by liquid chromatography coupled to on-line post-column derivatization. *Food Chem.* **361**, 173. 10.1016/j.foodchem.2021.130173 (2021). 34062455

[29] Han, L., Liu, S. G., Liang, J. Y., Li, N. B. & Luo, H. Q. Free-label dual-signal responsive optical sensor by combining resonance Rayleigh scattering and colorimetry for sensitive detection of glutathione based on ultrathin MnO2 nanoflakes. *Sens. Actuators B Chem.* **288**, 195–201. 10.1016/J.SNB.2019.02.117 (2019).

[30] No, H. S., Kim, T. & Hong, J. I. Iridium(Ⅲ) complex-based phosphorescent and electrochemiluminescent dual sensor for selective detection of glutathione. *Sens. Actuators B Chem.* **342**, 129868. 10.1016/J.SNB.2021.129868 (2021).

[31] Mu, L. et al. Simultaneous synthesis of carbon quantum dots and hydrothermal biochar from corn straw through hydrothermal treatment. *Ind. Crops Prod.* **219**, 119026. 10.1016/J.INDCROP.2024.119026 (2024).

[32] Baishya, N., Bora, N., Athparia, M., Padhi, P. & Kataki, R. Hydrothermal conversion of biomass for co-production of carbon quantum dots and biofuels. *Environ. Sci. Pollut. Res.* **32**, 24231–24245. 10.1007/S11356-024-35842-X (2025). 39751683

[33] Kirubanithy, K., Santhanam, A., Arunkumar, P. & Khan, M. M. Biomass derived nitrogen-doped water soluble carbon dots from Prosopis juliflora for Fe3 + sensing. *Microchem. J.* **212**, 113286. 10.1016/j.microc.2025.113286 (2025).

[34] Kathiravan, A., Ilango, B. & Jhonsi, M. A. Sustainable fluorescent carbon dots derived from sprouted palmyra seeds and their utilization in Fe3 + and ferritin detection in aqueous medium. *Mater. Today Commun.* **44**, 111862. 10.1016/J.MTCOMM.2025.111862 (2025).

[35] Chen, H., Huang, G., Li, Y. & Huang, Y. In situ green synthesis of Rosa sterilis-based carbon dots for Fe3 + fluorescent sensing in baijiu. *Food Biosci.* **62**, 105403. 10.1016/J.FBIO.2024.105403 (2024).

[36] Ren, H. et al. Transforming bio-waste lignin into amine functionalized carbon quantum dots for selective detection of trace Cu2 + in aqueous system. *Int. J. Biol. Macromol.* **273**, 133118. 10.1016/j.ijbiomac.2024.133118 (2024). 38871106

[37] Deng, X. Y. et al. Synthesis of functionalized carbon quantum dots as fluorescent probes for detection of Cu2+. *Chin. J. Anal. Chem.* **48**, e20126–e20133. 10.1016/S1872-2040(20)60054-8 (2020).

[38] Xue, J. J., Gan, M. H., Lu, Y. G. & Wu, Q. L. Fluorescence color tuning of dual-emission carbon quantum dots produced from biomass and their use in Fe3 + and Cu2 + detection. *New. Carbon Mater.* **39**, 1213–1226. 10.1016/S1872-5805(24)60869-3 (2024).

[39] Javeria, H., Xia Du, Z., Sharafat, S. & Abbas, M. Q. Eco-friendly paper-based dual-mode sensor from peanut waste for ultra-sensitive monitoring of Co2 + and Cu2 + ions. *J. Environ. Chem. Eng.* **14**, 122019. 10.1016/J.JECE.2026.122019 (2026).

[40] Simões, E. F. C., da Silva, J. C. G. E. & Leitão, J. M. M. Carbon dots from tryptophan doped glucose for peroxynitrite sensing. *Anal. Chim. Acta*. **852**, 174–180. 10.1016/J.ACA.2014.08.050 (2014). 25441895

[41] Ay, T., Wg, A. & Agyemang-Duah, E. Quantifying the albumin content of 14 tropical maize varieties in Ghana. *Int. J. Nutr. Food Sci.* **6**, 1. 10.19080/NFSIJ.2018.06.555680 (2018).

[42] Ananth, D. A. et al. Antibacterial potential of rutin conjugated with thioglycolic acid capped cadmium telluride quantum dots (TGA-CdTe QDs). *Spectrochim Acta Mol. Biomol. Spectrosc.* **138**, 684–692. 10.1016/J.SAA.2014.11.082 (2015). 25544184

[43] Raphela-Choma, P. P., Lukhwareni, R., Simelane, M. B. C., Motadi, L. R. & Choene, M. S. Antitumor effect of Iso-mukaadial acetate on MCF-7 breast cancer mice xenograft model. *Sci. Rep.* **14**, 1–12. 10.1038/S41598-024-64474-X;SUBJMETA (2024). 38877067 PMC11178819

[44] Kowalska-Krochmal, B. & Dudek-Wicher, R. The minimum inhibitory concentration of antibiotics: methods, interpretation, clinical relevance. *Pathogens* **10**, 165. 10.3390/PATHOGENS10020165 (2021). 33557078 PMC7913839

[45] Liang, H., Liu, H., Tian, B., Ma, R. & Wang, Y. Carbon quantum Dot@Silver nanocomposite–based fluorescent imaging of intracellular superoxide anion. *Microchim. Acta*. **187**, 8. 10.1007/S00604-020-04359-8 (2020). 32757083

[46] Mishra, M. K. & Singh, S. P. *Synthesis and Characterization of CQDs from Carbon Soot of Almond Oil and It’s Hierarchical Arrangement in 6CHBT LC Media: Role of Functionalization*. 10.21203/RS.3.RS-5049999/V1 (2024).

[47] Saini, S. et al. Sustainable synthesis of biomass-derived carbon quantum dots and their catalytic application for the assessment of α,β-unsaturated compounds. *RSC Adv.* **12**, 32619. 10.1039/D2RA05201F (2022). 36425689 PMC9661692

[48] Chen, Y. et al. *Two-Dimensional MXene Enabled Carbon Quantum Dots@Ag with Enhanced Catalytic Activity Towards the Reduction of p-Nitrophenol*. 10.1039/d1ra09177h (2022). PMC8981249 35425493

[49] Kechagias, A. et al. Development and characterization of N/S-carbon quantum dots by valorizing greek crayfish food waste. *Appl. Sci. (Switzerland)*. **13**, 730. 10.3390/APP13158730 (2023).

[50] Chandramohan, U. M. & Joseph, D. Hirshfeld surface, fukui function, molecular docking, molecular dynamics investigation on human immunodeficiency virus-1 (HIV) organism with 2,6-dibromo-4-chloroaniline. *Results Chem.* **11**, 101815. 10.1016/J.RECHEM.2024.101815 (2024).

[51] Singh, J., Inbaraj, B. S., Kaur, S., Rasane, P. & Nanda, V. Phytochemical analysis and characterization of corn silk (Zea mays, G5417). *Agronomy* **12**, 777. 10.3390/AGRONOMY12040777 (2022).

[52] Elkun, S., Ghali, M., Sharshar, T. & Mosaad, M. M. Green synthesis of fluorescent N-doped carbon quantum dots from castor seeds and their applications in cell imaging and pH sensing. *Sci. Rep.* **14**, 1–14 (2024). 39537758 10.1038/s41598-024-78745-0 PMC11560954

[53] Elkun, S., Ghali, M., Sharshar, T. & Mosaad, M. M. Green synthesis of fluorescent N-doped carbon quantum dots from castor seeds and their applications in cell imaging and pH sensing. *Sci. Rep.* **14**, 1–14 (2024). 39537758 10.1038/s41598-024-78745-0 PMC11560954

[54] Ahn, J., Pealer, C. & Kim, S. Analysis of carbon dots synthesized at different hydrothermal carbonization temperatures and their ferric ion detection performances. *Phys. Status Solidi B Basic. Res.* **261**, 345. 10.1002/pssb.202300345 (2024).

[55] Linh, N. V. et al. Effects of dietary corn silk (Zea mays L.) on growth, immune and antioxidant pathways, histological morphology, gut microbiome, and sensitivity to acute ammonia exposure in the koi carp (Cyprinus carpio var. koi). *Comp. Biochem. Physiol. B Biochem. Mol. Biol.* **279**, 111127. 10.1016/J.CBPB.2025.111127 (2025). 40633607

[56] Singh, J., Inbaraj, B. S., Kaur, S., Rasane, P. & Nanda, V. Phytochemical analysis and characterization of corn silk (Zea mays, G5417). *Agronomy* **12**, 777. 10.3390/AGRONOMY12040777 (2022).

[57] Tao, D. J. et al. Vitamin B9 derived nitrogen-doped graphene for metal-free aerobic oxidation of biomass-derived chemicals. *Green. Energy Environ.* **7**, 1084–1092. 10.1016/J.GEE.2021.01.008 (2022).

[58] Choppadandi, M. et al. Structural features regulated photoluminescence intensity and cell internalization of carbon and graphene quantum dots for bioimaging. *Mater. Sci. Eng., C*. **129**, 112366. 10.1016/J.MSEC.2021.112366 (2021). 34579885

[59] Wang, B. et al. Carbon dots in bioimaging, biosensing and therapeutics: a comprehensive review. *Small Sci.* **2**, 12. 10.1002/SMSC.202200012 (2022). PMC11935858 40213786

[60] Falke, S. & Betzel, C. *Dynamic Light Scattering (DLS) Principles, Perspectives, Applications to Biological Samples Abbreviations and Acronyms*. 10.1007/978-3-030-28247-9_6 (2019).

[61] Anilkumar, P. et al. Crosslinked carbon dots as ultra-bright fluorescence probes. *Small* **9**, 545–551. 10.1002/SMLL.201202000 (2013). 23413239

[62] Khan, M. J. et al. Ultrafast magneto-inductive synthesis of carbon dots from plant-based precursors using deep eutectic solvents: A comparative study with traditional hydrothermal methods. *Process Saf. Environ. Prot.* **195**, 106752. 10.1016/J.PSEP.2025.01.006 (2025).

[63] Ahn, J., Pealer, C. & Kim, S. Analysis of carbon dots synthesized at different hydrothermal carbonization temperatures and their ferric ion detection performances. *Phys. Status Solidi B Basic. Res.* **261**, 345. 10.1002/pssb.202300345 (2024).

[64] Jing, H. H., Adnan, M., Patel, M. & Sasidharan, S. Reproducibility roadblocks and standardization in carbon dot synthesis: a critical review of current practices, challenges, and future directions. *Mikrochim Acta*. **192**, 1. 10.1007/S00604-025-07683-Z (2025). 41269331

[65] Lu, X., Wang, F., Lei, W. & Xia, M. The synthesis and modification of highly fluorescent carbon quantum dots for reversible detection of water-soluble phosphonate-1-hydroxyethane-1,1-diphosphonic acid by fluorescence spectroscopy. *Luminescence* **36**, 200–209. 10.1002/BIO.3935;CTYPE:STRING:JOURNAL (2021). 32805085

[66] Xiong, Y. et al. Synthesis of up-conversion fluorescence N-doped carbon dots with high selectivity and sensitivity for detection of Cu2 + ions. *Cryst. (Basel)*. **13**, 812. 10.3390/cryst13050812 (2023).

[67] Wu, X. et al. Nitrogen-doped carbon quantum dots for fluorescence detection of Cu2 + and electrochemical monitoring of bisphenol A. *RSC Adv.* **8**, 20000–20006. 10.1039/c8ra03180k (2018). 35541682 PMC9080772

[68] Chandra, S. et al. Synthesis of highly fluorescent nitrogen and phosphorus doped carbon dots for the detection of Fe3 + ions in cancer cells. *Luminescence* **31**, 81–87. 10.1002/BIO.2927;ISSUE:ISSUE:DOI (2016). 25964146

[69] Mu, P., Han, Y. & Wang, J. Gram-scale green-emission carbon quantum dots produced from wood via the hydrothermal synthesis method for the detection of Fe (III). *Appl. Sci.* **15**, 1958. 10.3390/APP15041958 (2025).

[70] Srivastava, S. et al. Green synthesis of coconut coir-based carbon dots for efficient detection of ferric ions. *RSC Adv.* **15**, 41537–41545. 10.1039/D5RA03499J (2025). 41181014 PMC12573079

[71] Zulfajri, M., Gedda, G., Chang, C. J., Chang, Y. P. & Huang, G. G. Cranberry beans derived carbon dots as a potential fluorescence sensor for selective detection of Fe3 + ions in aqueous solution. *ACS Omega*. **4**, 15382–15392 (2019). 31572837 10.1021/acsomega.9b01333 PMC6761680

[72] Ahn, J., Song, Y., Kwon, J. E., Woo, J. & Kim, H. Characterization of food waste-driven carbon dot focusing on chemical structural, electron relaxation behavior and Fe3 + selective sensing. *Data Brief.* **25**, 104038. 10.1016/J.DIB.2019.104038 (2019). 31194181 PMC6554359

[73] Wu, F. et al. Facile synthesis of sulfur-doped carbon quantum dots from vitamin B1 for highly selective detection of Fe3 + ion. *Opt. Mater. (Amst)*. **77**, 258–263. 10.1016/j.optmat.2018.01.048 (2018).

[74] Preethi, M., Viswanathan, C. & Ponpandian, N. Fluorescence quenching mechanism of P-doped carbon quantum dots as fluorescent sensor for Cu2 + ions. *Colloids Surf. Physicochem Eng. Asp*. **653**, 9942. 10.1016/j.colsurfa.2022.129942 (2022).

[75] Zong, J. et al. Carbon dots as fluorescent probes for off-on detection of Cu2 + and l-cysteine in aqueous solution. *Biosens. Bioelectron.* **51**, 330–335. 10.1016/j.bios.2013.07.042 (2014). 23994615

[76] Karami, S., Shamsipur, M. & Barati, A. Intrinsic dual-emissive carbon dots for efficient ratiometric detection of Cu2 + and aspartic acid. *Anal. Chim. Acta*. **1144**, 26–33. 10.1016/j.aca.2020.11.032 (2021). 33453794

[77] Ali, H. R. H. et al. Development of dual function polyamine-functionalized carbon dots derived from one step green synthesis for quantitation of Cu2 + and S2 – ions in complicated matrices with high selectivity. *Anal. Bioanal. Chem.* **412**, 1353–1363. 10.1007/S00216-019-02362-4 (2020). 31900540

[78] Yang, J., Guo, Z. & Yue, X. Preparation of carbon quantum dots from corn straw and their application in Cu2 + detection. *Bioresources* **17**, 604–615. 10.15376/BIORES.17.1.604-615 (2022).

[79] Zheng, W. X. et al. Preparation of multi-colored carbon dots via pH-controlled degradation of wheat bran/o-phenylenediamine for Fe3 + ion detection. *RSC Adv.* **15**, 12028–12041. 10.1039/d4ra09117e (2025). 40248139 PMC12004230

[80] Jiang, C. et al. Presence of photoluminescent carbon dots in Nescafe^®^ original instant coffee: Applications to bioimaging. *Talanta* **127**, 68–74. 10.1016/J.TALANTA.2014.01.046 (2014). 24913858

[81] Mandani, S., Dey, D., Sharma, B. & Sarma, T. K. Natural occurrence of fluorescent carbon dots in honey. *Carbon N Y*. **119**, 569–572. 10.1016/J.CARBON.2017.04.075 (2017).

[82] Dong, Y. et al. Natural carbon-based dots from humic substances. *Sci. Rep.* **5**, 1–8. 10.1038/srep10037 (2015). PMC4421865 25944302

[83] Sk, M. P., Jaiswal, A., Paul, A., Ghosh, S. S. & Chattopadhyay, A. Presence of amorphous carbon nanoparticles in food caramels. *Sci. Rep.* **2**, 1–5. 10.1038/srep00383 (2012). PMC3337553 22540029

[84] Yang, H., Ran, G., Yan, J., Zhang, H. & Hu, X. A sensitive fluorescence quenching method for the detection of tartrazine with acriflavine in soft drinks. *Luminescence* **33**, 349–355. 10.1002/BIO.3420 (2018). 29094465

[85] Guo, B., Liu, G., Hu, C., Lei, B. & Liu, Y. The structural characteristics and mechanisms of antimicrobial carbon dots: a mini review. *Mater. Adv.* **3**, 7726–7741. 10.1039/D2MA00625A (2022).

[86] Wang, X., Hu, C., Gu, Z. & Dai, L. Understanding of catalytic ROS generation from defect-rich graphene quantum-dots for therapeutic effects in tumor microenvironment. *J. Nanobiotechnol.* **19**, 340. 10.1186/s12951-021-01053-6 (2021). PMC8547047 34702276

[87] Vantangoli, M. M., Madnick, S. J., Huse, S. M., Weston, P. & Boekelheide, K. MCF-7 human breast cancer cells form differentiated microtissues in scaffold-free hydrogels. *PLoS ONE*. **10**, 135426. 10.1371/JOURNAL.PONE.0135426 (2015). PMC4534042 26267486

[88] Pirsko, V. et al. An effect of culture media on epithelial differentiation markers in breast cancer cell lines MCF7, MDA-MB-436 and SkBr3. *Medicina* **54**, 11. 10.3390/MEDICINA54020011 (2018). PMC6037242 30344242

