# Supplementary material for: Green production of multi-functional carbon dots from agricultural corn silk waste: application in heavy metal (Fe3+/Cu2+) fluorometric sensing and in vitro cytotoxicity
Source: Sci Rep. 2026 Aug 31;16:27517. doi: 10.1038/s41598-026-65469-6 (PMC13538382; doi:10.1038/s41598-026-65469-6)
Supplement: Supplementary file 1 [file 41598_2026_65469_MOESM1_ESM.docx]

**Green Production of Multi-functional Carbon Dots from Agricultural Corn Silk Waste: Application in Heavy Metal (Fe^3+^ /Cu^2+^) Fluorometric Sensing and *In Vitro* Cytotoxicity**

Dina Abdelmonsef ^a^, Sherif Hammad ^b^, Mohsen Ghali ^a*^

^a^ *School of Basic and Applied Sciences, Egypt-Japan University of Science and Technology (E-JUST), New Borg El-Arab City, Alexandria, Egypt*

^b^ *PharmD Program, Egypt-Japan University of Science and Technology (E-JUST), New Borg El-Arab City, Alexandria, Egypt*

**^Corresponding author.^ *^E-mail address:^*** [**^mohsen.ghali@ejust.edu.eg^**](mailto:mohsen.ghali@ejust.edu.eg)

**Table of contents**

**Fig S1.** Dynamic light scattering (DLS) of non-synthetic CDs and synthetic CDs prepared at 160,200 and 240 °C.

**Fig S2.** (a)The PL intensities of CDs and CDs + Cu^2+^ at 400 nm within the pH range 2.0–11.0. (b) Relationship between quenching efficiency and pH.

**Fig S3.** Effect of interaction time on the fluorescence intensity of the synthesized CDs at different Cu²⁺ concentrations (0.48 and 1.0 μM).

**Fig S4.** Photoluminescence emission spectra of three independently prepared batches of non-synthetic CDs (a) and CDs synthesized at 200 °C (b), demonstrating excellent batch-to-batch reproducibility (n=3).

**Fig S5.** Photostability of the non-synthetic CDs under continuous 400 nm illumination for 240 min.

**Fig S6.** (a, c) Fluorescence emission spectra of CDs before and after the addition of different metal ions under identical conditions. (b, d) Relative fluorescence intensity (F/F₀) of CDs-160 °C and CDs-240 °C in the presence of various metal ions.

**Fig S7.** UV-vis spectra of non-synthetic CDs (a) and CDs-200 ͦ C (b) in the absence and presence of Cu^2+^ and Fe^3+^ ions.

**Fig S8.** (a) FT-IR spectra of non-synthetic and (b) CDs-200 ͦ C with and without Cu^2+^ Fe^3+^ ions.

**Fig S9.** (a) Fluorescence emission spectra of CDs at different Fe^3+^ concentrations in bottled water, and (b) the linear relationship between F_0_/F and Fe³⁺concentration.

**Fig S10.** Fluorescence intensity of F/ F_0_ against mixed metal ions in non-synthetic CDs.

**Fig S11.** Fluorescence intensity of F/ F_0_ against mixed metal ions in CDs-200 ͦ C.

Fig S1. Dynamic light scattering (DLS) of non-synthetic CDs and synthetic CDs prepared at 160,200 and 240 °C.

Fig S2. (a)The PL intensities of CDs and CDs + Cu^2+^ at 400 nm within the pH range 2.0–11.0. (b) Relationship between quenching efficiency and pH.

Fig S3. Effect of interaction time on the fluorescence intensity of the synthesized CDs at different Cu²⁺ concentrations (0.48 and 1.0 μM).

Fig S4. Photoluminescence emission spectra of three independently prepared batches of non-synthetic CDs (a) and CDs synthesized at 200 °C (b), demonstrating excellent batch-to-batch reproducibility (n=3).

Fig S5. Photostability of the non-synthetic CDs under continuous 400 nm illumination for 240 min.

Fig S6. (a, c) Fluorescence emission spectra of CDs before and after the addition of different metal ions under identical conditions. (b, d) Relative fluorescence intensity (F/F₀) of CDs-160 °C and CDs-240 °C in the presence of various metal ions.

Fig S7. UV-vis spectra of non-synthetic CDs (a) and CDs-200 ͦ C (b) in the absence and presence of Cu^2+^ and Fe^3+^ ions.

The UV–vis absorption spectra of CDs in the presence of metal ions exhibited a significant increase in absorbance across both the ultraviolet and visible regions. The characteristic absorption peak (∼260–280 nm) showed a pronounced enhancement upon metal ion addition. The non-synthetic CDs + Cu^2+^ system displayed a noticeably broader absorption band compared to CDs see Fig S7a, whereas the synthetic CDs at 200 ͦ C + Fe^3+^ system exhibited a blue shift accompanied by peak broadening as shown in Fig S7b. These spectral changes indicate the formation of ground-state complexes and/or the emergence of new electronic transitions arising from interactions between the CDs and the metal ions.[13–15]

Fig S8. (a) FT-IR spectra of non-synthetic and (b) CDs-200 ͦ C with and without Cu^2+^ Fe^3+^ ions.

The FT-IR spectrum of the non-synthetic CDs exhibited noticeable changes upon the addition of Cu^2+^ ions, suggesting the possible formation of a CDs/ Cu^2+^coordination complex (Fig S8a). The decrease in the intensities of the bands at 3261 and 1184 cm⁻¹ may be attributed to the interaction of Cu^2+^ with –NH₂ / – OH and C–N functional groups on the CDs surface. Additionally, the reduced absorbance of the C–H vibrations in the CDs/ Cu^2+^ system indicates that the surface chemical environment of the CDs was influenced by the presence of Cu^2+^ ions, likely due to their affinity toward nitrogen-containing groups[10].in case of CDS-200 °C, the FTIR spectra (Fig S8a) reveal a noticeable shift and enhancement of the band around 1770 cm⁻¹, attributed to C=O stretching[11], indicating the interaction of Fe^3+^ ions with surface carbonyl groups of the CDs. Additionally, changes in the intensity of O–H/N–H, C=N, and C–O–C bands further support the involvement of both oxygen- and nitrogen-containing functional groups. These spectral variations suggest a multi-site coordination mechanism and confirm the formation of a CD + Fe^3+^ complex [12].

Fig S9. (a) Fluorescence emission spectra of CDs at different Fe^3+^ concentrations in bottled water, and (b) the linear relationship between F_0_/F and Fe³⁺concentration.

The selectivity of non-synthetic CDs and CDs-200 °C toward Cu²⁺ (Fig. S10) and Fe³⁺ (Fig. S11) ions, respectively, was evaluated in the presence of common interfering metal ions. The fluorescence of both systems was only slightly affected by other ions, whereas Cu²⁺ and Fe³⁺ induced pronounced quenching. In mixed-ion systems, the fluorescence response remained dominated by Cu²⁺ and Fe³⁺, confirming the high selectivity of each sensor.

Fig S10. Fluorescence intensity of F/ F_0_ against mixed metal ions in non-synthetic CDs

Fig S11. Fluorescence intensity of F/ F_0_ against mixed metal ions in CDs-200 ͦ C.
